# The Recombinant Inhibitor of DNA Binding Id2 Forms Multimeric Structures via the Helix-Loop-Helix Domain and the Nuclear Export Signal

**DOI:** 10.3390/ijms19041105

**Published:** 2018-04-07

**Authors:** Cornelia Roschger, Mario Schubert, Christof Regl, Ancuela Andosch, Augusto Marquez, Thomas Berger, Christian G. Huber, Ursula Lütz-Meindl, Chiara Cabrele

**Affiliations:** 1 Department of Biosciences, University of Salzburg, Billrothstrasse 11 and Hellbrunner Strasse 34, 5020 Salzburg, Austria; cornelia.roschger@sbg.ac.at (C.R.); mario.schubert@sbg.ac.at (M.S.); christof.regl@sbg.ac.at (C.R.); ancuela.andosch@sbg.ac.at (A.A.); c.huber@sbg.ac.at (C.G.H.); ursula.luetz-meindl@sbg.ac.at (U.L.-M.); 2 Christian Doppler Laboratory for Innovative Tools for Biosimilar Characterization, University of Salzburg, Hellbrunner Strasse 34, 5020 Salzburg, Austria; 3 Department of Chemistry and Physics of Materials, University of Salzburg, Jakob-Haringer Strasse 2a, 5020 Salzburg, Austria; augusto.marquez@imb-cnm.csic.es (A.M.); thomas.berger@sbg.ac.at (T.B.)

**Keywords:** helix-loop-helix domain, intrinsic disorder, protein oligomerization, protein self-assembly, inhibitor of DNA binding, protein fibrils

## Abstract

The inhibitor of DNA binding and cell differentiation 2 (Id2) is a helix-loop-helix (HLH) protein that acts as negative dominant regulator of basic-HLH transcription factors during development and in cancer. The structural properties of Id2 have been investigated so far by using synthetic or recombinant fragments reproducing single domains (N-terminus, HLH, C-terminus): the HLH domain tends to dimerize into a four-helix bundle, whereas the flanking regions are flexible. In this work, the intact protein was expressed in *E. coli*, solubilized from inclusion bodies with urea, purified and dissolved in water at pH~4. Under these conditions, Id2 was obtained with both cysteine residues disulfide-bonded to β-mercaptoethanol that was present during the solubilization process. Moreover, it existed in a self-assembled state, in which the N-terminus remained highly flexible, while the HLH domain and, surprisingly, part of the C-terminus, which corresponds to the nuclear export signal (NES), both were involved in slowly tumbling, rigid structures. The protein oligomers also formed twisted fibrils that were several micrometers long and up to 80 nm thick. These results show that self-assembly decreases the backbone flexibility of those two protein regions (HLH and NES) that are important for interaction with basic-HLH transcription factors or for nucleocytoplasmic shuttling.

## 1. Introduction

The inhibitors of DNA binding and cell differentiation (Id1 to Id4) represent a small class of mammalian proteins within the large family of the helix-loop-helix (HLH) transcription factors [1]. In contrast to the other members of the family, the four Id proteins do not interact with DNA due to the lack of a DNA-binding motif. The Id proteins contain a highly similar HLH domain that is required for the dimerization with a DNA-binding basic-HLH (bHLH) transcription factor; such heterodimer is assumed to form a parallel four-helix bundle, similar to the one found in crystals of DNA-bound bHLH homodimers (e.g., E47 [2], MyoD [3], and Max [4]). However, in contrast to bHLH-bHLH dimers, Id-bHLH dimers are unable to form a complex with the DNA. Thus, by sequestering bHLH transcription factors in non-DNA-binding dimers, the Id proteins act as negative regulators of bHLH-mediated gene expression. Besides bHLH transcription factors, the Id proteins are also able to interact with proteins that do not contain an HLH domain: these include the subunit of the 26S proteasome (S5a) [5], the retinoblastoma protein (pRb) [6,7], the winged-helix-turn-helix transcription factors Elk-1 and SAP-1/2 [8], the anaphase-promoting complex/cyclosome (Apc/C) subunits Apc1, Apc5, Apc8/Cdc23 [9] and the von-Hippel Lindau (VHL)-elongin C complex [10]. Some of these interactions are specific for individual Id-family members, which suggests that they are not only mediated by the HLH Id motif, but also by the N-terminal and C-terminal regions. For example, Iavarone and Lasorella have recently shown that the Id2 protein binds the VHL-elongin C complex with the N-terminal segment 14–34 [10]. Interestingly, this interaction is prevented upon phosphorylation of the Id2 residue Thr-27, which underlines the importance of the N-terminal Id2 region to control protein–protein interactions.

In addition to heteroassociation with bHLH and non-bHLH proteins, both homo- and hetero-dimerization have been observed within the Id family: for example, Id2 [11] and Id3 [12] can form homodimers, whereas Id4 can interact with Id1-3 [13,14]. The multiple specificity of the Id proteins in molecular recognition events is likely to reflect the lack of a well-defined monomeric structure [15,16]. However, whereas a significant amount of biological data on the Id proteins has been published, structural and conformational data on these proteins are still limited. In particular, to the best of our knowledge, only the Id3 protein has been investigated so far as intact protein by circular dichroism (CD) spectroscopy [17]. For the other Id proteins, structural investigation has been carried out on the separated domains: for example, CD studies of synthetic peptides reproducing the HLH, N-terminal and C-terminal regions of the four Id proteins have been reported [18,19,20,21,22]. Also, one crystal structure and one NMR solution structure of the homodimer of the HLH domain of Id2 [23] and Id3 [24], respectively, are known. The HLH Id domain has been observed in the folded state only as a dimer [23,24] or part of high-order oligomers [20,21], while the N-terminal and C-terminal regions display no preferred conformation [21]. Our previous investigation on synthetic polypeptides reproducing the full-length Id3 protein as well as N-terminally truncated analogs suggests that the N-termini and C-termini do not significantly contribute to the helical conformation of the full-length protein [17], which in turn delimitates the localization of secondary structure mostly within the HLH domain. Based on the fact that disordered regions coexist with a region that is able to fold upon self- or hetero-association, we have recently suggested that the Id proteins feature properties that are characteristic of intrinsically disordered (or unstructured) proteins (IDPs) [25]. IDPs adopt no stable secondary and tertiary structure, but rather an ensemble of interconverting conformations over a significant number of sequential residues [26,27].

Herein, we report on the results of a spectroscopic and biophysical investigation of recombinant full-length human Id2 protein obtained from *E. coli* inclusion bodies, which shed light on the self-assembly behavior of the protein and its tendency to adopt multimeric structures.

## 2. Results and Discussion

### 2.1. Mass Spectrometry of Recombinant Id2 Protein

The human Id2 protein was expressed in *E. coli* and isolated from inclusion bodies upon solubilization with 8 M urea in the presence of 2% β-mercaptoethanol (βME). The solubilized protein was purified under denaturing conditions (0.05% TFA in water/acetonitrile) by semi-preparative HPLC, lyophilized and, finally, characterized by analytical HPLC, western blot analysis, and mass spectrometry (Figures S1–S3). The MALDI-TOF mass spectra of the lyophilized recombinant protein obtained from independent preparations revealed a different degree of protein homogeneity, which was caused by different levels of oxidation of cysteine and methionine residues. One sample contained three major species corresponding to the unmodified protein and two modified species containing one or two mixed disulfide bridges with βME (Figure S3A,B). Apparently, the reducing conditions used during the isolation of the protein from the *E. coli* inclusion bodies were not sufficiently strong to avoid thiol oxidation. Additionally, each of these species coexisted with a variant incorporating at least one oxidized methionine, Met(O). In another sample, in addition to the species having the two Cys/βME disulfide bridges, high-order oligomers of the type Id2_n_βME_n_, with *n* = 2–4, were detected. Based on the number of free cysteine residues present in the self-associated forms, it is interesting to note that, whereas dimerization might be caused by disulfide-bond formation between the two free cysteine residues, trimerization and tetramerization (or association of two dimers) must be based also on non-covalent interactions (Figure S3C). The most homogeneous sample, which was used for further investigation and is referred to as Id2’ in the following subsections, contained both cysteine residues disulfide-bonded to βME, and one oxidized methionine residue (Figure S3D).

### 2.2. CD Spectroscopy

**Figure 1:**
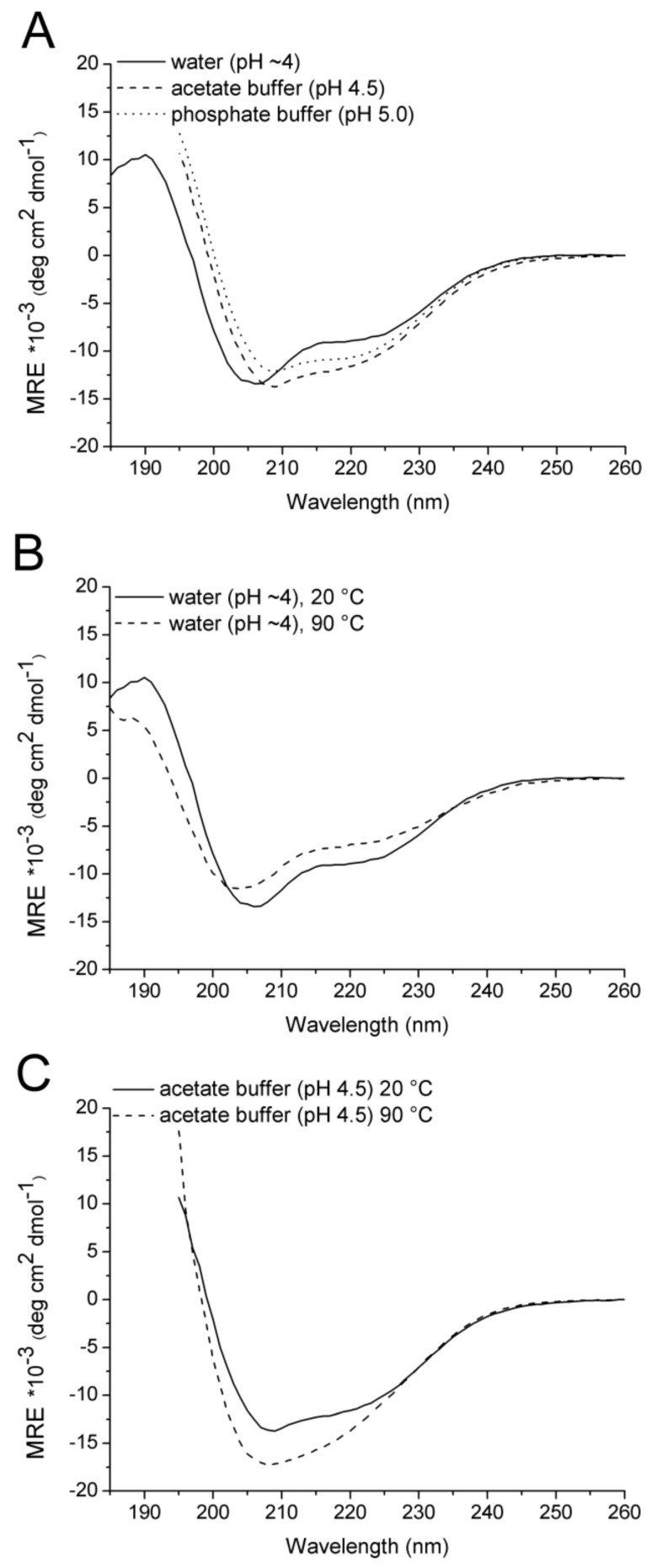
Circular dichroism (CD) spectra of recombinant Id2’ (30 μM). (**A**) CD curves in 100 mM buffer (pH 4.5 or 5.0) or in pure water (pH~4). (**B**) CD curves in pure water at 20 °C and 90 °C. (**C**) CD curves in sodium acetate buffer at 20 °C and 90 °C. The CD spectra of the buffered solutions could be recorded only until 195 nm due to exceeding detector voltage below this wavelength. The CD unit is the molar residue ellipticity (MRE = [θ]_R_) that was divided by 10^3^ for convenience of representation of the Y-axis.

In previous CD studies on Id2 protein fragments, we showed that the N-terminal and C-terminal regions have poor propensity to build secondary structure elements [19,21]. In contrast, the HLH domain has high helical content, which results from the intra- and inter-molecular packing of two short helices (H1 and H2), as shown in the crystal structure of the HLH Id2 homodimer (PDB ID: 4AYA [23]). To compare the CD signature of the HLH Id2 domain with that of the full-length Id2 protein, we performed CD measurements of the recombinant protein sample Id2’. As previous CD studies of the HLH Id2 domain were made in phosphate buffer at pH 7.3, we tried first to dissolve Id2’ in the same buffer. Unfortunately, the protein was poorly soluble at neutral pH. Thus, we performed a buffer screening to determine the pH-dependent solubility of the protein (Table S1). We found that Id2’ was soluble in non-buffered water (pH~4) as well as in sodium acetate buffer (pH 4.5–5.0), phosphate buffer (pH 5.0–5.7), and 2-(N-morpholino)ethanesulfonate (MES) buffer (pH 5.8–6.2). Therefore, we measured the CD spectra of Id2’ in water, sodium acetate buffer (pH 4.5) and phosphate buffer (pH 5.0). As shown in Figure 1A, all three CD spectra indicate the presence of ordered structure; however, the CD intensity at the wavelengths characteristic of α-helix (about 208 nm and 222 nm) and β-sheet (about 216 nm) is significantly different between the non-buffered and buffered protein solutions, which indicates a different contribution of the single secondary structure elements to the overall conformation. We performed the deconvolution of the CD spectrum only for the sample in pure water, which could be recorded until 180 nm and, thus, it provides a more robust secondary structure prediction [28,29,30,31] (Table 1). In contrast, the buffered samples could be measured only until 195 nm, due to exceeding detector voltage below this wavelength. Although we did not predict the secondary structure composition in buffer due to lack of data below 195 nm, the CD difference spectra clearly show that a CD component appears in buffer, which may be attributed to the gain of β-strands, as suggested by the negative band around 216 nm and a positive contribution below 207–209 nm (Figure S4A, inset).

Upon heating to 90 °C, both the buffered and non-buffered protein solutions still contained a significant amount of secondary structure (Figure 1B,C). However, whereas the CD spectra of the non-buffered solution at 20 °C and 90 °C were similar in shape while having different intensity, the CD spectrum of the buffered solution at 90 °C showed a broad minimum in place of the two minima around 208 and 220 nm. Again, due to the lack or low quality of the CD data below 195 nm for the buffered sample, we applied the deconvolution method only to the CD spectrum of the non-buffered sample (Table 1). Comparison of the secondary structure compositions predicted at 20 °C and 90 °C reveals a temperature-induced partial transition of α-helix conformation into disordered or irregular conformation. Accordingly, the CD component, which disappeared upon the heating of the sample in pure water, was characteristic of an α-helix (Figure S4B, inset), in agreement with the predicted loss of α-helical elements at 90 °C. In contrast, the CD component, which appeared upon the heating of the sample in buffer, was characteristic of β-strands and, probably, of aggregated structures (Figure S4C, inset). The different response to the heating of the protein in pure water and in buffer is in agreement with the observation that the overall conformation of the protein is not identical in non-buffered and buffered solutions, resulting in different thermal transitions. It should be added that the changes of secondary structure, which were induced by increasing the temperature to 90 °C, were reversible upon cooling only in the case of the non-buffered solution (Figure S5).

**Figure 2:**
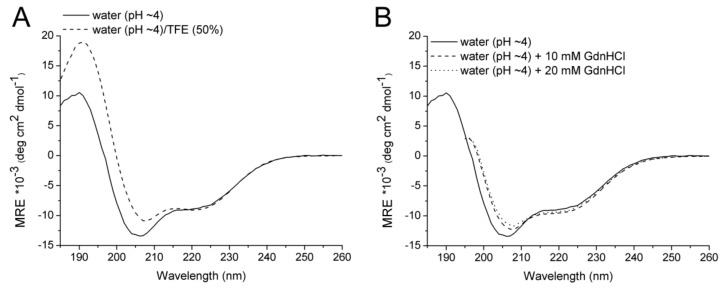
CD spectra of recombinant Id2’ (30 μM). (**A**) CD curves in pure water (pH~4) or in water/TFE (1:1, *v*/*v*). (**B**) CD curves in water (pH~4) without or with addition of millimolar concentrations of GdnHCl. The CD spectra of the solutions containing GdnHCl could be recorded only until 195 nm due to exceeding detector voltage below this wavelength. The CD unit is the molar residue ellipticity (MRE = [θ]_R_) that was divided by 10^3^ for convenience of representation of the Y-axis.

Furthermore, we investigated the effect of 2,2,2-trifluoroethanol (TFE) on the non-buffered protein conformation of Id2’. TFE is known to stabilize the secondary structure of peptides, mainly by water displacement and consequent induction of intramolecular hydrogen bonds [32]. The CD curve of the sample in water containing 50% TFE could be recorded until 180 nm and, thus, it was deconvoluted [28,29,30,31] (Table 1). Comparison of the predicted secondary structure composition in the absence and in the presence of 50% TFE revealed a moderate TFE-mediated stabilization of the α-helix and β-sheet components at the expenses of disordered and irregular components. This fits well with the fact that the CD spectrum in the presence of TFE has less negative CD contribution below 210 nm, which indicates a reduction of disordered and irregular conformation (Figure 2A). However, the negative band around 220 nm, which is highly characteristic of an α-helix, has the same intensity in the absence and in the presence of TFE; this also fits well with the predicted secondary structure composition that indicates only a moderate stabilization of the α-helical conformation by TFE (32% in 50% TFE vs. 28% in pure water).

In a previous study, we observed that submolar concentrations of guanidine hydrochloride (GdnHCl) increased the helical content of the synthetic HLH domain of the Id2 protein [21]. Indeed, besides the denaturing effect of GdnHCl at molar concentrations, its stabilizing effect at submolar concentrations has been also reported [33]. This is attributed to the fact that the guanidinium cation may interact with cation-binding sites of a folded or partially folded protein, thus stabilizing the structure [34]. Based on this previous observation, we measured the effect of 10 mM and 20 mM GdnHCl on the conformation of the non-buffered protein: interestingly, we obtained an effect comparable to that of TFE (Figure 2B), consisting of the less negative and about 2 nm red-shifted minimum for the π–π* || electronic transition as well as of the about 2 nm red-shifted cross point from negative to positive CD intensities. This indicates that submolar GdnHCl contributes to a moderate stabilization of the secondary structure elements of Id2’. 

Based on the CD measurements, the Id2’ protein is predicted to contain ordered regions covering ~60% of the sequence, as estimated by deconvolution [28,29,30,31] of the CD spectrum in pure water (Table 1). A helix content of about 28% for the 134-residue long Id2 protein corresponds to about 38 residues, which would closely match the length of the two helices of the HLH domain in the crystal structure of the HLH Id2 homodimer [23]. Moreover, this helix content is comparable to that found previously for the synthetic full-length Id3 protein by CD spectroscopy [17]. This suggests that most of the contribution to the helix content arises from the HLH domain. However, the presence of helical motifs in the HLH domain has been correlated so far with the propensity of this domain to self-associate: for example, the formation of dimers and tetramers of the HLH domain of Id1 has been proposed on the basis of CD and analytical ultracentrifugation measurements [20]. Moreover, homodimeric structures of the HLH domains of Id2 [23] and Id3 [24] have been determined by X-ray crystallography and NMR spectroscopy, respectively. Likewise, HLH homodimers have been identified for DNA-bound bHLH transcription factors by X-ray crystallography, as it is case of the bHLH domains of E47 [2] and MyoD [3].

In summary, the CD measurements of the lyophilized Id2’ protein in acidic aqueous solutions showed that the protein was ~60% ordered, with the α-helix content being in agreement with the helical regions observed in the crystal structure of the HLH Id2 homodimer [23]. However, the overall conformation was sensitive to the buffer condition, the chaotropic salt GdnHCl, the secondary structure-stabilizing TFE, and the temperature. In particular, acetate (pH 4.5) and phosphate (pH 5.0) buffers not only favored the β-sheet component of the protein, but they also facilitated the protein misfolding upon heating, which prevented a reversible transition during the cooling phase. 

### 2.3. NMR Spectroscopy

To gain more insights into the secondary structure content, we prepared ^13^C,^15^N-labeled Id2 protein for the investigation by NMR spectroscopy, which is referred to as ^13^C,^15^N-Id2’’ (or simply Id2’’) in the following. High-resolution ESI-MS analysis confirmed a high degree of isotopic labeling (~96%), and also showed that both cysteine residues were involved in mixed disulfide bonds with βME, as proved by intact protein MS with and without reductive treatment with TCEP, as well as by LC-ESI-MS/MS of the protein digested with the endoproteinase Glu-C (Figures S6 and S7). The latter measurement revealed also the presence of partial oxidation of methionine at position 1. 

**Figure 3:**
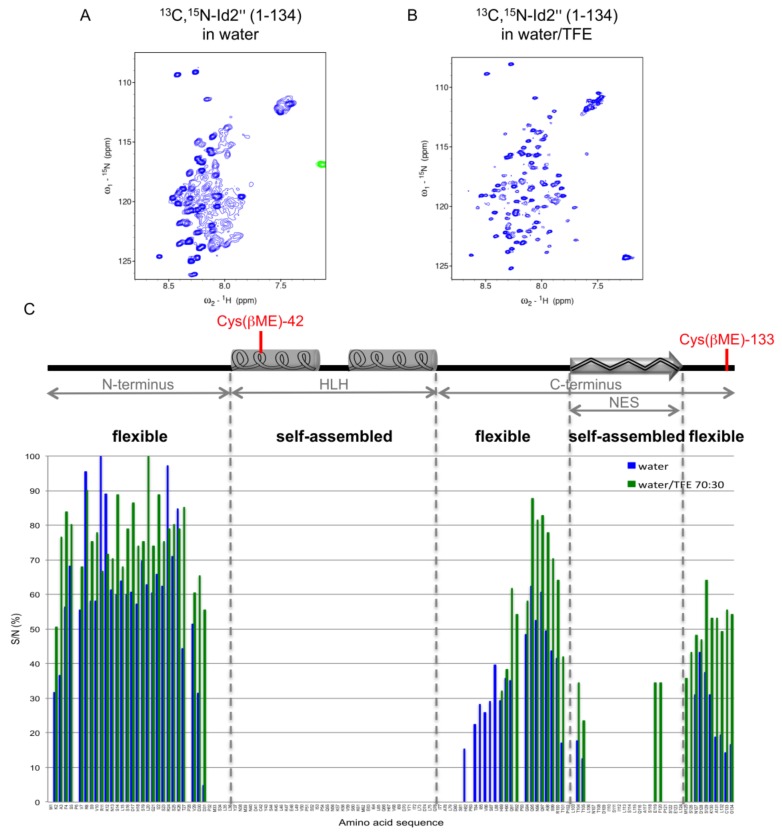
^1^H-^15^N Heteronuclear single quantum correlation (HSQC) spectra of ^13^C,^15^N-Id2’’ in water without or with 30% TFE, at the concentration of 0.86 and 0.5 mM, respectively. (**A**) The spectrum in water (with 7% D_2_O) was measured at 313 K. (**B**) The spectrum in water/TFE (with 7% D_2_O) was measured at 298 K. (**C**) The S/N ratio of the ^15^N-H crosspeaks is given in percentage with respect to the highest S/N ratio in each spectrum (Arg-11 or Leu-20 for the spectrum in water or water/TFE, respectively). Chemical shift assignment (ppm) is reported in Tables S2 and S3.

NMR measurements of the ^13^C,^15^N-labeled protein were performed in water or water/TFE 70:30 (Figure 3). Intriguingly, only the N-terminus and part of the C-terminus could be assigned, whereas the HLH sequence and the central region of the C-terminus were not visible or gave severely broadened signals. The intensity profile of the ^15^N-H crosspeaks of a ^15^N-HSQC spectrum in absence and presence of 30% TFE was similar; however, in the presence of TFE, the signal-to-noise (S/N) ratio of some signals was superior, and residues 92, 119, 120, 125, and 126 could be additionally assigned. In contrast, residues 82–88, which were visible in water, disappeared in water/TFE. It is interesting to note that the two NMR-undetectable regions match very well self-aggregation prone regions of the Id2 protein: indeed, besides the ability of the HLH domain to self-assemble into helix bundles, we previously reported that the central region of the C-terminus (residues 103–124), which contains the nuclear export signal (NES) responsible for the Id2 shuttling from the nucleus to the cytoplasm [35], forms β-sheet rich aggregates and even nanofibrils [36].

The fact that the HLH domain and the NES region are broadened or non-visible in the NMR spectra suggests that they are involved in high-order oligomers with slow tumbling. To prove this, we performed a diffusion-ordered spectroscopy (DOSY) experiment with the unlabeled protein Id2’, which confirmed the presence of large oligomers consisting of about 16 monomers on average (Figure S8).

To perform the complete NMR assignment of the ^13^C,^15^N-Id2’’ protein backbone, it was necessary to work under denaturing conditions. Therefore, we measured the NMR sample of the ^13^C,^15^N-labeled protein in H_2_O containing 8 M urea at pH 2.3 (Figure S9). Complete sequence specific assignment of the protein backbone was achieved (Table S4). Due to the similarity of the chemical shifts under denaturing conditions, the typical triple-resonance experiments relying on ^13^Cα and ^13^Cβ for linking spin systems sometimes fail. However, this lack of dispersion could be compensated by an (H)NCANH experiment [37] that displayed excellent dispersion of both ^15^N dimensions even under denaturing conditions (Figure S10). In addition, amino acid-type selective ^15^N-HSQC spectra [38] further supported the sequential backbone assignment (Figure S11). 

We compared the ^13^Cα and ^13^Cβ chemical shifts of the Id2’’ sample in 8 M urea at pH 2.3 with the random coil (RC) chemical shifts from three different reference libraries: (a) the library from Schwarzinger et al., which is based on GGXGG peptides in 8 M urea at pH 2.3, including neighbor correction for the ^13^Cα resonances [39,40]; (b) the library from Wishart et al., which is based on GGXAGG or GGXPGG peptides in 1 M urea at pH~5 [41]; (c) the ncIDP library from Tamiola et al., which contains neighbor-corrected RC shifts extrapolated from 14 IDPs [42]. The Id2’’ RC chemical shifts were in general in good agreement with all three libraries (|Δδ| < 0.5 ppm), confirming the denatured state of the Id2’’ protein under these conditions (Figures S12 and S13).

**Figure 4:**
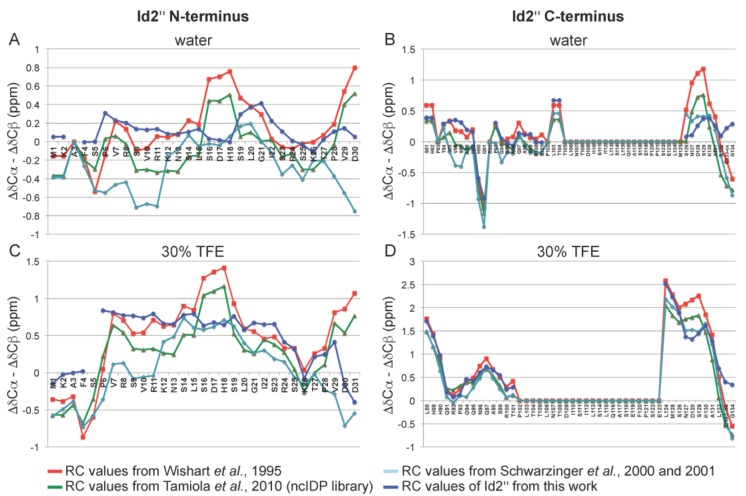
Secondary chemical shifts (deviations of the chemical shifts from the RC values) of ^13^C,^15^N-Id2’’ in water at the concentration of 0.86 mM (**A** and **B**), and water/TFE (70:30, *v*/*v*) at the concentration of 0.5 mM (**C** and **D**). The reference random coil (RC) values were from Wishart et al. (obtained from GGXAGG or GGXPGG in 1 M urea at pH~5) [41], Schwarzinger et al. (obtained from GGXGG in 8 M urea at pH 2.3 and neighbor corrected for ^13^Cα) [39,40], Tamiola et al. (obtained from IDPs and neighbor corrected, except for Cys-ox, which were from Wishart et al. [41]) [42], and this work (obtained from the Id2’’ sample in 8 M urea at pH 2.3). The secondary chemical shifts were smoothed by a three-point function [44].

Further, we used the three RC chemical shift libraries as well as the Id2’’ RC chemical shifts obtained in this work to calculate the secondary chemical shifts (deviations of the chemical shifts from the RC values) for the N-terminal and C-terminal regions measured in water and water/TFE (70:30, *v*/*v*). Figure 4 shows the profiles of the differences between Δδ^13^Cα and Δδ^13^Cβ as obtained for each RC chemical shift library (the corresponding histograms can be found in Figures S14 and S15). All four profiles are in good agreement and confirm the major flexible character of the N-terminus as well as of the C-terminus (except the NES sequence). However, it is interesting to note that they also indicate the presence of weakly populated α-helix-like regions, whose presence becomes more evident in a secondary structure stabilizing environment like in 30% TFE, in particular for residues 7–25, 88–101 and 124–132 (see also the effect of TFE on a synthetic peptide reproducing the N-terminal Id2 region 1–35, as shown by CD spectroscopy in Figure S16). This suggests that the N-terminus and the C-terminal parts flanking the NES region of Id2’’ feature a highly dynamic conformation with the intrinsic propensity to fold into transient secondary structure motifs ideally suited to adapt to different binding partners [43].

In summary, the NMR investigation of the lyophilized Id2’’ protein in pure water and in the presence of 30% TFE showed that the protein existed in a self-associated form (16mers on average), which prevented the structure elucidation of the HLH and NES regions. In contrast, by evaluating the secondary chemical shifts of the N-terminus as well as of the C-terminal parts flanking the NES sequence, these regions appeared to be highly flexible, without well-defined secondary structure but with the ability to form transient secondary structure elements in a suitable environment.

### 2.4. Dynamic Light Scattering

**Figure 5:**
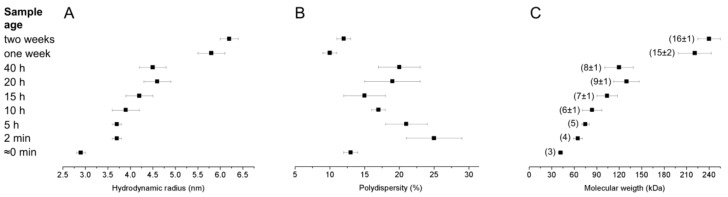
DLS measurements of a filtered Id2’ solution in pure water (80 μM) over a period of two weeks at 21 °C. The hydrodynamic radius (**A**), polydispersity (**B**), and molecular weight (**C**) corresponding to each sample age represent the averaged value of 7–10 acquisitions. The number of self-associated Id2’ monomers is reported in the brackets in panel C.

As shown by the NMR investigation, the Id2’’ protein forms high-order oligomers obtained by the assembly of about 16 monomers on average containing two slowly tumbling, rigid domains (HLH and NES) as well as flanking regions with high degree of flexibility. To further look at these soluble aggregates, we performed dynamic light scattering (DLS) measurements of a filtered Id2’ sample in water at the concentration of 80 μM over a period of two weeks. The results are summarized in Figure 5: the hydrodynamic radius was about 3 nm in the sample that was measured immediately after filtration, but it reached about 6 nm in a two weeks old sample. The polydispersity values show that the size distribution in the samples measured immediately after filtration and after one or two weeks was narrower than in the intermediate samples. As polydispersity below 20% is attributed to monodisperse solutions [45], these three samples can be considered monodisperse (polydispersity below 15%), whereas the intermediate samples have more polydispersity character. The estimated molecular weight of the soluble aggregates ranged from about 42 kDa for the freshly filtered sample to about 240 kDa for the two weeks old sample, which would correspond to the association of three to 16 monomers. The latter value fits quite well with the one obtained by the DOSY experiment (Figure S8). Based on the change of the hydrodynamic radius, size distribution and molecular weight over a period of two weeks, the self-assembly of Id2’ into large oligomers seems to be an isodesmic (stepwise association) rather than a cooperative process, with the formation of intermediate oligomeric states. Recently, it has been reported that hen-eggwhite lysozyme at alkaline pH, a model to investigate protein aggregation, also forms multimeric aggregates by an isodesmic aggregation pathway [46].

To the best of our knowledge, the formation of soluble high-order oligomers of HLH transcription factors has been reported so far only for MyoD, for which a micellization process involving more than 100 monomers has been proposed [47]. Our data on the self-assembly of the Id2’ and Id2’’ samples into multimeric structures support the idea that proteins, whose function relies on transient interactions with different proteins [48], as it is the case for IDPs, might exploit the formation of multimeric structures as a kind of reservoir in equilibrium with the monomeric species. It should be mentioned that self-association of peptides and proteins for storage purpose is known to occur, for example, in secretory granules, where large aggregates, including amyloid-like (cross β-sheet rich) ones, have been observed [49,50,51,52,53].

### 2.5. ATR-FTIR Spectroscopy

**Figure 6:**
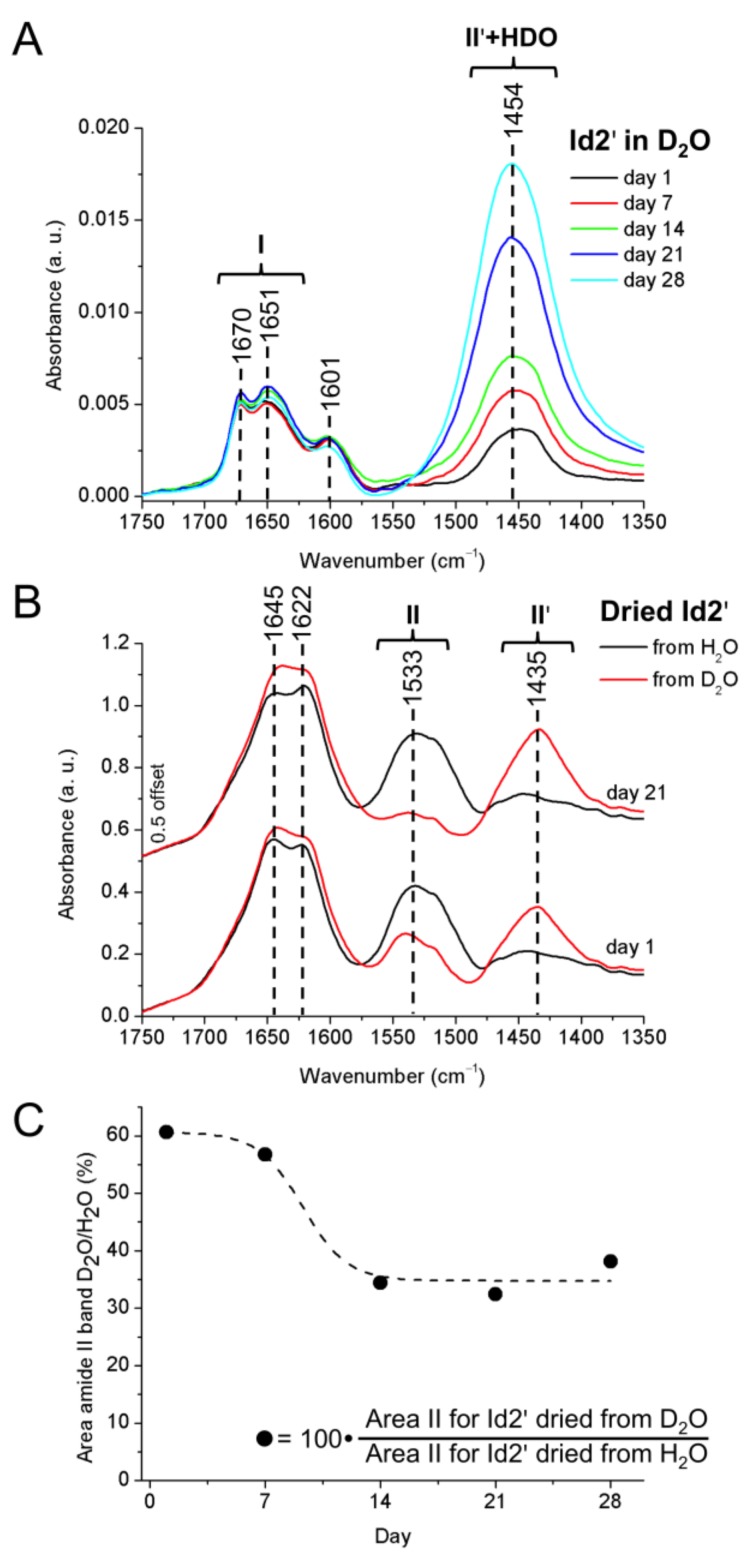
Attenuated total reflection (ATR)-FTIR spectra of Id2’ upon aging. (**A**) Spectra of a 1 mM protein solution in D_2_O recorded over a period of 28 days (sample storage at 5 °C). (**B**) Spectra of Id2’ films obtained upon concentration to dryness under a slow nitrogen stream for 2.5 h of 100 μL of a 1 mM Id2’ solution in D_2_O or H_2_O. (**C**) Impact of aging on amide hydrogen isotope exchange. The extent of deuterium-to-hydrogen exchange was estimated from the amide II band areas in the spectra of Id2’ films dried from D_2_O and H_2_O, respectively.

Attenuated total reflection (ATR)-FTIR spectra of recombinant Id2’ were measured both in D_2_O (Figure 6A) and H_2_O (Figure S17). In the amide I region of the deuterated sample, which was measured shortly after preparation (indicated as day 1 in Figure 6), two main bands are present and may be attributed, respectively, to turns (1670 cm^−1^), and α-helix plus disordered structures (1651 cm^−1^). In addition, the contribution of the deuterated Arg and His side chains may be assigned at 1601 cm^−1^ [54,55]. To estimate the relative contribution of different conformational elements to the amide I band, we applied the curve-fitting approach that is based on the second derivative and deconvolution of the experimental curve [55].

The analysis yielded contributions of ~73% α-helix plus disordered structure (1650 cm^−1^), ~12% β-sheets (1616 cm^−1^) and ~14% turns (1674 cm^−1^). Although a contribution of deuterated Asn and Gln side chains to the amide I band is expected (~1650 cm^−1^ and 1635–1654 cm^−1^ for deuterated Asn and Gln, respectively [54]), the estimated secondary structure content is in fair agreement with the results from CD spectroscopy (Table 1). The amide II band, which is located at 1541 cm^−1^ in H_2_O (Figure S17), is shifted to 1454 cm^−1^ in D_2_O (amide II’ band), due to the complete deuteration of the protein backbone. Aging of the protein solution at 5 °C for up to 28 days did not result in any significant change of the amide I band. In contrast, we observed a strong increase in the absorbance over the amide II’ band region 1400–1500 cm^−1^. This contribution was associated with the formation of HDO upon hydrogen isotope exchange of D_2_O with water vapor during sample storage and handling. However, such an increase in the HDO content in the solvent did not lead to a detectable back-protonation of the protein, as reflected by the invariance of protein specific bands (Figure 6A).

Furthermore, we examined Id2’ protein films that were deposited on the ATR prism by drying 100 μL of protein solution in D_2_O in a slow nitrogen stream for 2.5 h. The IR spectra of the protein films differed significantly from the solution spectra (Figure 6B). On the one hand, the IR band centered at 1601 cm^−1^ and attributed to the deuterated side chains of Arg and His [54] (Figure 6A) was absent after sample drying. In addition, the amide II band at 1533 cm^−1^ and the amide II’ band at 1435 cm^−1^ coexisted in the spectra of the protein films (Figure 6B). These results indicate that the protein was partially back-protonated upon drying. The absence of the band at 1601 cm^−1^ in the dried films indicates the complete hydrogen isotope exchange (deuterium to hydrogen) for Arg and His side chains. These ionizable side chains are assumed to be always water exposed, independent of the size and stability of the oligomers, which results in a rapid exchange of deuterium with hydrogen during the drying process due to contact with water vapor in the environment. The situation is different for protected amide groups in the structured and self-assembled regions of the protein. Indeed, we observed only a partial hydrogen isotope exchange (deuterium to hydrogen) of the backbone amides for all samples (Figure 6B,C). Interestingly, the hydrogen isotope exchange was less prominent for those samples that were at least 14 days old. This might correlate with a reduced water exposure of the protein backbone, which might arise from an increased order of oligomerization and/or stability of the multimeric structures upon aging of the protein sample.

### 2.6. Transmission Electron Microscopy

**Figure 7:**
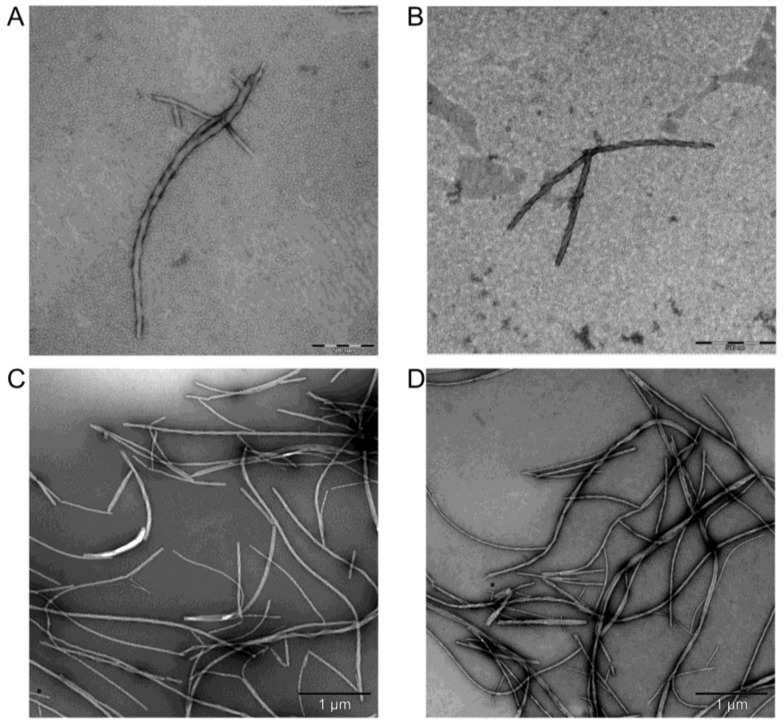
TEM images of Id2’ fibrils. The protein solution in pure water (1.2 mM) was kept at 5 °C for a period of four weeks. Images of aliquots diluted to 50 μM and negatively stained with uranyl acetate after 16 h (**A** and **B**: scale bar = 500 nm) and four weeks (**C** and **D**: scale bar = 1 μm) are shown.

In a previous study we showed that a synthetic peptide containing the NES sequence of Id2 aggregates and forms fibrils that may reach the thickness of 10 nm and the length of 1 μm after four weeks [36]. As also the recombinant full-length protein preparations of the unlabeled (Id2’) and ^13^C,^15^N-labeled (Id2’’) samples showed high propensity to self-aggregate, we investigated, whether this may lead to the formation of ordered nanostructures. For this purpose, we recorded transmission electron microscopy (TEM) images of negatively stained samples prepared with water solutions of Id2’, which were stored at 5 °C for 16 h or four weeks (Figure 7). Both measurements detected twisted fibrils that were several micrometers long and up to 80 nm thick. More and longer fibrils could be found in the four weeks old sample, indicating that fibril formation was independent from the preparation protocol for the TEM measurement, but it was rather dependent on the self-assembly status of the protein in the solution.

## 3. Conclusions

In this work, recombinant human Id2 protein was obtained from *E. coli* inclusion bodies under denaturing conditions and characterized by biophysical methods. Protein samples from independent preparations showed different homogeneity, which was due to the oxidation of cysteine and methionine residues. The most homogeneous sample contained both Cys-42 and Cys-133 disulfide bonded to βME, which was used during the solubiliziation process with urea. This protein sample was found to be soluble at pH values below 7. CD spectra in water (pH~4) and in sodium acetate buffer (pH 4.5) showed slightly different conformations that behaved, however, in a fully different manner upon increasing the temperature: indeed, only the non-buffered protein underwent a reversible transition that was associated with moderate loss/complete regain of α-helix. Thus, the secondary structure composition of the protein was sensitive to the environment. NMR and DLS measurements indicated the presence of oligomeric structures of the protein in aqueous solution, which were characterized by the coexistence of rigid and flexible regions. Interestingly, the slowly tumbling, rigid regions comprised both the HLH sequence, which represents the dimerization domain of all HLH transcription factors, and the NES motif, which is responsible for the nuclear-cytoplasmic shuttling of Id2. Time-dependent DLS measurements support an isodesmic mechanism of association, at least for the larger oligomers (more than four subunits). In contrast, the formation of dimers, trimers and tetramers must occur immediately after dissolution of the protein in water. Similar to other aggregation-prone proteins, Id2’ was also able to build mature twisted fibrils. 

In conclusion, the biophysical characterization of the recombinant Id2 samples reported here shows that this protein forms multimers containing specific regions with low or high backbone flexibility, which raises the question, whether this feature will be of relevance for the stability and activity of the protein in the cell. Indeed, Id2 regions like the HLH domain, which is required for protein–protein interactions with bHLH transcription factors, and, surprisingly, the NES motif, which regulates the protein subcellular localization via nuclear export receptors, were found to be included in slowly tumbling, rigid structures. Further studies will be necessary, which will need to contemplate also alternative expression protocols under native conditions, and self-assembly studies in a cellular system.

## 4. Materials and Methods

### 4.1. Materials

The full-length human Id2 cDNA sequence (NCBI accession no. NM_002166) in the pEX-A vector and the different primers were from Eurofins MWG (Ebersberg, Germany). Bacto Agar and Difco LB Broth Miller (Luria Bertani) were from Becton, Dickinson and Company (Sparks, MD, USA). The pET22b(+) vector was from Novagen (Merck, Darmstadt, Germany). FastBreak^TM^ cell lysis reagent and endoproteinase Glu-C (protease V8) were from Promega (Mannheim, Germany). Acetic acid, ampicillin, acetonitrile, agarose, bovine serum albumin, guanidine hydrochloride (GdnHCl), *E. coli* Dh5α competent cells, *E. coli* BL21 (DE3) chemically competent cells, ethanol, isopropyl-β-d-thiogalactopyranoside (IPTG), deoxyribonuclease I (DNAse I) from bovine pancreas, triton X-100, phenylmethylsulfonyl fluoride (PMSF), (ethylenedinitrilo)tetraacetic acid (EDTA), sodium chloride, 2-amino-2-(hydroxymethyl)-1,3-propanediol hydrochloride (TRIS-HCl), 2,2,2-trifluoroethanol (TFE), urea, uranyl acetate, and D_2_O (99.9% atom %D, for ATR-FTIR measurements) were from Sigma-Aldrich (Merck, Darmstadt, Germany). Lysozyme was from Appli Chem (Darmstadt, Germany). β-Mercaptoethanol (βME), TRIS buffered saline, tween-20, and colorimetric AP substrate reagent kit were from Biorad (Hercules, CA, USA). Trifluoroacetic acid (TFA) was from Alfa Aesar (Thermo Fisher, Karlsruhe, Germany). α-Cyano-4-hydroxycinnamic acid was from Acros Organics (Fisher Scientific, Vienna, Austria). Polyclonal rabbit antibody against human Id2 and alkaline phosphatase conjugated anti-rabbit secondary antibody were from Thermo Fisher Scientific (Waltham, MA, USA). d-Glucose (U-^13^C_6_, 99% atom %D) and ammonium chloride (^15^N, 99% atom %D) were from Cambridge Isotope Laboratories, Inc. (Andover, MA, USA). D_2_O and 2,2,2-trifluoroethanol-d_3_ (TFE-d_3_) for NMR measurements were from ARMAR Chemicals (Leipzig, Germany). 

### 4.2. Methods

#### 4.2.1. Construction of the pET Expression Plasmids Encoding Human Id2 1–134

The coding region of full-length human Id2 1**–**134 (NCBI accession no. NM_002166) was purchased in the pEX-A vector (Eurofins MWG Operon). To exclude the His-tag of the pET22b(+) expression vector, the cDNA was designed with a double stop at the end of the sequence. The Id2_pEX-A plasmid was amplified with primers incorporating 5’ NdeI and 3’ XhoI sites (forward primer: AAA AAA CAT ATG AAA GCC TTT TCA CCG GTT C and reverse primer: AAA AAA CTC GAG TCA TCA CCC ACA GAG TGC TTT CG) and inserted into the multiple cloning site of the pET22b(+) expression vector. The plasmid was transformed into Dh5α competent cells and plated on LB-agar containing 100 mg/ml ampicillin. Colony PCR was performed and positive colonies were verified by sequencing (Eurofins MWG Operon).

#### 4.2.2. Expression of Unlabeled and ^13^C,^15^N-Labeled Recombinant Human Id2 1–134

The pET22b(+) Id2 1–134 expression plasmid was transformed into One Shot competent *E. coli* BL21 (DE3) cells and plated on a LB-agar plate containing 100 mg/mL ampicillin. One single colony was inoculated into 20 mL LB-medium containing 100 mg/mL ampicillin and grown overnight at 37 °C in an incubator under constant agitation. The next day, the overnight culture was added to 1 L fresh medium, and grown at 37 °C under constant agitation. After the OD600 reached 0.6–0.8, the recombinant inclusion body protein expression was obtained with 1 mM IPTG, followed by incubation at 37 °C under constant agitation for 4 h.

For the ^13^C,^15^N-labeled protein expression, a 50 mL overnight culture in LB-medium was gently centrifuged, and the cells were resuspended and cultivated in 1 L M9 minimal medium [56] containing 1 g/L ^15^NH_4_Cl, 4 g/L ^13^C-glucose and 100 mg/mL ampicillin at 37 °C under constant agitation. After the OD600 reached 0.6–0.8, the recombinant inclusion body protein expression was induced with 1 mM IPTG, followed by incubation at 37 °C under constant agitation for 4 h.

#### 4.2.3. Protein Purification

Cells were subsequently harvested by centrifugation (4000 rpm, 30 min at 4 °C) and lysed in FastBreak^TM^ cell lysis reagent, including 80 µL of 10 mg/mL lysozyme, 4 µL of 100 mM PMSF, 20 µL of 1 mg/mL DNase I, and 100 µL βME per 1 g wet cell pellet. After 30 min incubation under constant agitation, followed by 30 min centrifugation at 4° C and 4000 rpm, the pellet was washed with wash buffer I (50 mM TRIS-HCl, 10 mM EDTA, 100 mM NaCl, 0.05% triton X-100, pH 8.0). After centrifugation (4000 rpm, 30 min at 4 °C), a second wash step was performed with wash buffer II (50 mM TRIS-HCl, 1 mM EDTA, 100 mM NaCl, pH 8.0). The full-length human Id2 protein from the inclusion bodies was solubilized with 8 M urea containing 2% βME and purified via semi-preparative HPLC by using a Thermo Fisher Scientific (Germering, Germany) equipment (model Dionex™ UltiMate™ 3000) and a NUCLEOSIL^®^ C-18 (250 × 10 mm, 5 µm) column from Macherey-Nagel (Düren, Germany). The mobile phase consisted of the following binary system: (A) 0.06% TFA in water, and (B) 0.05% TFA in acetonitrile. The gradient was 10% B for 3 min, 10–70% B over 40 min, with a flow rate of 5 mL/min, and UV detection at 220 nm. After lyophilization, the recombinant full-length protein was characterized by analytical HPLC, western blot analysis, MALDI-TOF-MS and ESI-MS (Figures S1–S3, S6 and S7). The analytical HPLC system (model Dionex™ UltiMate™ 3000) and the column (BioBasic-18, 250 × 4.6 mm, 5 µm) were both from Thermo Fisher Scientific. The gradient was 10% B for 5 min, 10–70% B over 30 min, with a flow rate of 1 mL/min (mobile phase and UV detection were the same as used for the semi-preparative runs).

#### 4.2.4. Mass Spectrometry

MALDI-TOF mass spectra were recorded on an Autoflex mass spectrometer from Bruker Daltonics (Bremen, Germany) using α-cyano-4-hydroxycinnamic acid as matrix. LC-ESI mass spectra were recorded on a Dionex™ UltiMate™ 3000 Rapid Separation system coupled to a Q Exactive^TM^ benchtop quadrupole-Orbitrap instrument equipped with an Ion Max^TM^ source with heated electrospray ionization, all from Thermo Fisher Scientific. Chromatographic separation of the intact ^13^C,^15^N-Id2’’ protein sample with or without TCEP treatment was conducted at a flow rate of 200 μL/min employing a Discovery^®^ BIO Wide Pore C18 column (150 × 2.1 mm, 3 µm; Supelco Analytical), operated at a temperature of 50 °C. Mobile phases A and B were 0.05% TFA in water, and 0.05% TFA in acetonitrile, respectively. The gradient was 5% B for 2 min, 5–70% B over 10 min. UV detection was carried out at 214 nm with a 2.5 µL flow cell. The peptide fragments obtained after protein treatment with the protease Glu-C were chromatographically separated on the same system utilizing a Hypersil GOLD aQ C18 column (100 × 1.0 mm, 1.9 µm, 175 Å pore size, Thermo Fisher Scientific) at a flow rate of 60 μL/min operated at a temperature of 50 °C. The gradient was 2% B for 5 min, 2–35% B over 45 min. For mass spectrometry of the intact protein the instrument settings were as follows: source heater temperature of 250 °C, spray voltage of 4.0 kV, sheath gas flow of 15 arbitrary units, auxiliary gas flow of 5 arbitrary units, capillary temperature of 275 °C, S-lens RF level of 60.0, AGC target of 1e6 and a maximum injection time of 100 ms. Measurements were performed in full scan mode in a range of *m/z* 1000–3000 at a resolution of 140,000 at *m/z* 200 and averaging of ten microscans. For peptide mapping each scan cycle consisted of a full scan at a scan range of *m/z* 200–2000 at a resolution setting of 70,000, followed by five data-dependent HCD scans at 28 NCE at a resolution setting of 17,500.

Lyophilized recombinant protein was dissolved in water for MALDI-TOF-MS analysis or in 175 mM ammonium acetate (pH 6.8) for ESI-MS analysis. For the digestion with the endoproteinase Glu-C, ^13^C,^15^N-Id2’’ was treated with Glu-C (protein to protease ratio of 20:1) in 150 mM ammonium bicarbontate (pH 7.9) for 1 h at 37 °C. The Glu-C-digested protein was analyzed by LC-ESI-MS/MS.

#### 4.2.5. Buffer Screening

The pH screening was set up using the hanging drop method with a 24-well tissue culture plate and siliconized glass cover slips, as described previously [57]. The buffer (1 mL, 100 mM) was pipetted into each reservoir. A 2 µL drop of recombinant protein (Id2’) in water at a concentration of 0.27 mM was pipetted onto each cover slip. To each 2 µL drop, 1 µL of the 100 mM reservoir buffer was added and gently mixed. The cover slips were inverted and sealed onto the wells with petroleum jelly. The plate was incubated for 24 h at 37 °C and additionally five days at 21 °C. The degree of protein solubility was assessed along the equilibration process by scoring the presence of precipitate in the drops immediately after preparation as well as after 15 min, 24 h and six days (score 0–5: 0, no precipitate; 5, precipitate covering the drop area completely. See Table S1).

#### 4.2.6. CD Spectroscopy

The CD spectra were recorded on a Chirascan-plus ACD spectropolarimeter (Applied Photophysics, Leatherhead, UK) using a 1 mm quartz cuvette at 20 °C. The temperature-scan experiments (20–90 °C) were performed with a 0.5 mm quartz cuvette. For each CD spectrum, four scans were accumulated using a step resolution of 0.1 nm, a bandwidth of 1 nm, a response time of 2 s, and a scan speed of 20 nm/min. The CD spectrum of the solvent was subtracted from that of the protein (Id2’) solution to eliminate interferences from the cell, solvent and optical equipment. The CD signal was normalized as mean-residue ellipticity (deg·cm^2^·dmol^−1^) by using the protein concentration obtained by UV measurements on an Agilent Cary 60 UV-Vis spectrophotometer (an extinction coefficient of 1480 M^−1^·cm^−1^ per Tyr residue [58] was applied).

#### 4.2.7. NMR Spectroscopy

The NMR spectra were recorded on a Bruker AVANCE III HD 600 MHz spectrometer equipped with a QXI (^1^H/^13^C/^15^N/^31^P)-quadruple resonance probe (Rheinstetten, Germany). Unless stated otherwise, NMR spectra were measured at 298 K. ^13^C,^15^N-Id2’’ protein samples were measured at a concentration of 0.86 mM in H_2_O/D_2_O (93:7, *v*/*v*), 0.5 mM in TFE/H_2_O/D_2_O (30:63:7, *v*/*v*), or 0.74 mM in 8 M urea with 5% D_2_O (pH 2.3, adjusted with HCl until reaching a final chloride concentration of 180 mM). Chemical shift assignment of the protein backbone was achieved using the standard triple resonance experiments: HNCACB, CBCA(CO)NH, HNCA, HNCO and HN(CA)CO [59]. In addition, an (H)NCANH experiment [37] was applied for the denatured sample, taking advantage of the high ^15^N dispersion in the ω1 dimension. Amino acid type-selective ^15^N-HSQC spectra using the MUSIC selection were recorded for Ala and Thr/Val/Ile/Ala [38]. ^1^H chemical shifts were referenced to 2,2-dimethyl-2-silapentane-5-sulfonic acid (DSS) using an external sample of 0.5 mM DSS/2 mM sucrose for the samples in water and water/TFE, or 1 mM DSS in 8 M urea at pH 2.3 for the denatured sample. ^13^C and ^15^N resonances were indirectly referenced using scaling factors of 0.251449530 and 0.101329118, respectively, according to Markley et al. [60]. Spectra were processed with Topspin 3.2 (Bruker Biospin, Rheinstetten, Germany) and analyzed with Sparky 3.115 [61]. The chemical shifts of the protein sample Id2’’ measured in urea have been deposited in the Biological Magnetic Resonance Data Bank (BMRB) with accession no. 27358. 2D DOSY experiments were measured with an unlabeled Id2’ protein sample at 0.27 mM concentration in H_2_O/D_2_O (94:6, *v*/*v*). The Bruker pulse sequence stebpgp1s19, with stimulated echo, bipolar gradient pulses, one spoil gradient and 3-9-19 water suppression, was used with a linear gradient (53.5 G/cm) stepped between 2% and 95%. For the calibration curve, protein standards were measured at concentrations ranging from 0.2 to 0.4 mM, which included aprotinin, lysozyme, carbonic anhydrase, ovalbumin and bovine serum albumin. Typical parameters were: td = 32, a gradient duration of δ = 1.0–1.4 ms, and an echo delay Δ between 200 and 400 ms.

#### 4.2.8. Dynamic Light Scattering

Dynamic light scattering (DLS) experiments were performed at 21 °C with a DynaPro NanoStar instrument (Wyatt Technology, Dernbach, Germany) using a 120-mW laser of 658 nm wavelength at 100% power and a 90° detection angle. Lyophilized recombinant protein (Id2’) was dissolved in water and filtered through a filter with 0.2 μm pore size (final protein concentration of the filtered solution: 80 µM). The protein sample was kept at 21 °C for two weeks. Aliquots were taken at different time points and measured. Each measurement consisted of 7–10 acquisitions of 10 s duration at the constant temperature of 21 °C. The collected data were analyzed by using the Dynamics 7.0.2 software (Wyatt Technology) with water as reference solvent, and the globular model for molecular weight estimation.

#### 4.2.9. ATR-FTIR Spectroscopy

For IR measurements, an attenuated total reflection (ATR) unit (PIKE Technologies, Veemax II, Madison, WI, USA) was coupled to a Bruker Vertex 70 FTIR spectrometer equipped with a MCT detector (Ettlingen, Germany). The measurements were performed at an incident angle of 45° using a hemispherical ZnSe prism. Lyophilized recombinant protein (Id2’) was dissolved in H_2_O or D_2_O at the concentration of 1 mM. The protein sample was kept at 5 °C for four weeks. Aliquots (100 μL) were taken at different time points and measured. After collecting the spectra of 100 µL sample for the liquid measurements, the sample was dried by applying a slow nitrogen stream for 2.5 h, and the spectra were recorded again. Spectra were obtained by averaging 100 scans at a resolution of 4 cm^−1^ and are represented as −log(R/R_0_), where R and R_0_ are the reflectance values corresponding to the single beam spectra recorded for the sample and reference, respectively. Reference spectra were those of pure H_2_O or D_2_O for wet samples, and of air for dried samples. Data analysis was performed with the program Origin (OriginLab Corporation, Northampton, MA, USA). To estimate the content of secondary structure, the amide I band in the spectra of the protein in D_2_O was deconvoluted following a procedure described previously [62]. Briefly, the second derivative of the experimental spectra (upon subtraction of the straight region over the range 1800–2000 cm^−1^) was used to identify the number and position of the overlapping components bands, whose single contribution to the experimental curves was obtained by the iterative Chi square minimization of the combination of Gaussian-type peaks using the Levenberg-Marquardt algorithm. 

#### 4.2.10. Transmission Electron Microscopy

Lyophilized recombinant protein (Id2’) was dissolved in water to a final concentration of 1.2 mM. The protein sample was kept at 5 °C for four weeks. An aliquot was taken after 16 h or after four weeks, it was diluted to 50 µM, and a 5 µL drop was transferred onto a formvar coated copper grid. After air-drying, the sample was negatively stained with 10 µL of a uranyl acetate solution (2% *w*/*v* in water). The images were recorded using a LEO 912AB Omega transmission electron microscope (Carl Zeiss, Oberkochen, Germany) at 120 kV acceleration voltage and filtered at zero energy loss.

## Figures and Tables

**Table 1:** Estimated secondary structure elements composition of recombinant Id2′ (30 μM), as obtained by deconvolution of the CD spectra ^1^.

Solvent	Temperature (°C)	α (%)	β (%)	Turn (%)	Disordered (%)
Water	20	28	12	20	40
Water/50% TFE	20	32	16	20	33
Water	90	19	14	15	52

^1^ The deconvolution has been performed by using the experimental CD curve from 180 to 260 nm, and the Contin-LL algorithm [28,29] with the reference set no. 6 [30] on Dichroweb (http://dichroweb.cryst.bbk.ac.uk/html/home.shtml) [31].

## Supplementary Materials

Supplementary materials can be found at http://www.mdpi.com/1422-0067/19/4/1105/s1.

## Abbreviations

ATR	Attenuated Total Reflection
CD	Circular Dichroism
DLS	Dynamic Light Scattering
HLH	Helix-Loop-Helix
Id2	Inhibitor of DNA Binding and Cell Differentiation 2
IDP	Intrinsically Disordered Protein
MCT	Mercury Cadmium Telluride
βME	β-Mercaptoethanol
TEM	Transmission Electron Microscopy
TFA	Trifluoroacetic Acid
