# Supplementary material for: The Recombinant Inhibitor of DNA Binding Id2 Forms Multimeric Structures via the Helix-Loop-Helix Domain and the Nuclear Export Signal
Source: Int J Mol Sci. 2018 Apr 7;19(4):1105. doi: 10.3390/ijms19041105 (PMC5979349; doi:10.3390/ijms19041105)
Supplement: Supplementary file 1 [file ijms-19-01105-s001.pdf]

Supplementary Materials

The recombinant inhibitor of DNA binding Id2 forms multimeric structures via the helix-loop-helix domain and the nuclear export signal

Cornelia Roschger [1], Mario Schubert [1,2], Christof Regl [1,2], Ancuela Andosch [1], Augusto Marquez [3], Thomas Berger [3], Christian G. Huber [1,2], Ursula Lütz-Meindl [1] and Chiara Cabrele [1,2,*]

[1] Department of Biosciences, University of Salzburg, Billrothstrasse 11 and Hellbrunner Strasse 34, 5020 Salzburg, Austria

[2] Christian Doppler Laboratory for Innovative Tools for Biosimilar Characterization, University of Salzburg, Hellbrunner Strasse 34, 5020 Salzburg, Austria

[3] Department of Chemistry and Physics of Materials, University of Salzburg, Jakob-Haringer Strasse 2a, 5020 Salzburg, Austria

[*] Correspondence: chiara.cabrele@sbg.ac.at

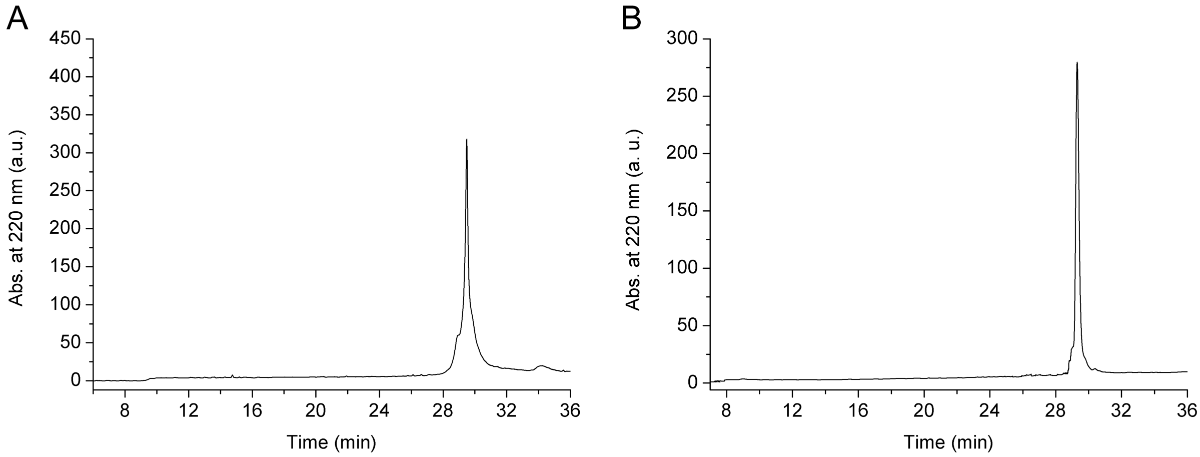

Figure S1. HPLC profiles of the lyophilized recombinant Id2 protein after purification by semi-preparative HPLC. (A) The Id2 sample contains oxidized species (oxidized Met and mixed disulfide bonds with βME). (B) The ^{13}C,^{15}N-Id2 sample contains the two mixed disulfide bonds of Cys-42 and Cys-133 with βME (see MS spectra below). Gradient: 10% B for 5 min, 10-70% B over 30 min, with A = 0.06% TFA in water, and B = 0.05% TFA in acetonitrile.

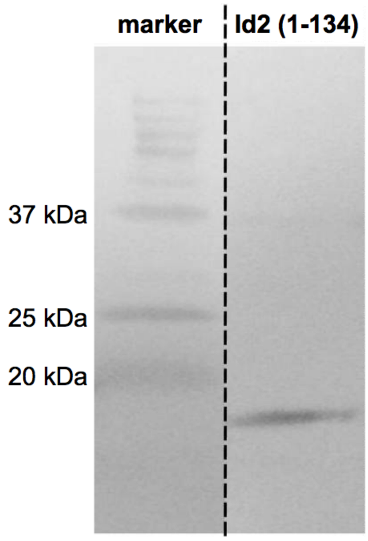

Figure S2. Western blot analysis of recombinant Id2 after HPLC purification. The sample was separated by SDS-PAGE after DTT reduction and transferred to a nitrocellulose membrane. After blocking the membrane with 5% BSA in TRIS buffered saline with 0.2 % tween-20 overnight at 4 °C, the Id2 protein was determined using polyclonal rabbit anti-Id2 antibody as the primary and alkaline phosphatase conjugated goat anti-rabbit immunoglobulin G (H+L) as the secondary antibody. Antibodies were incubated for 1 h at room temperature each. The bands were visualized using the colorimetric AP substrate reagent kit.

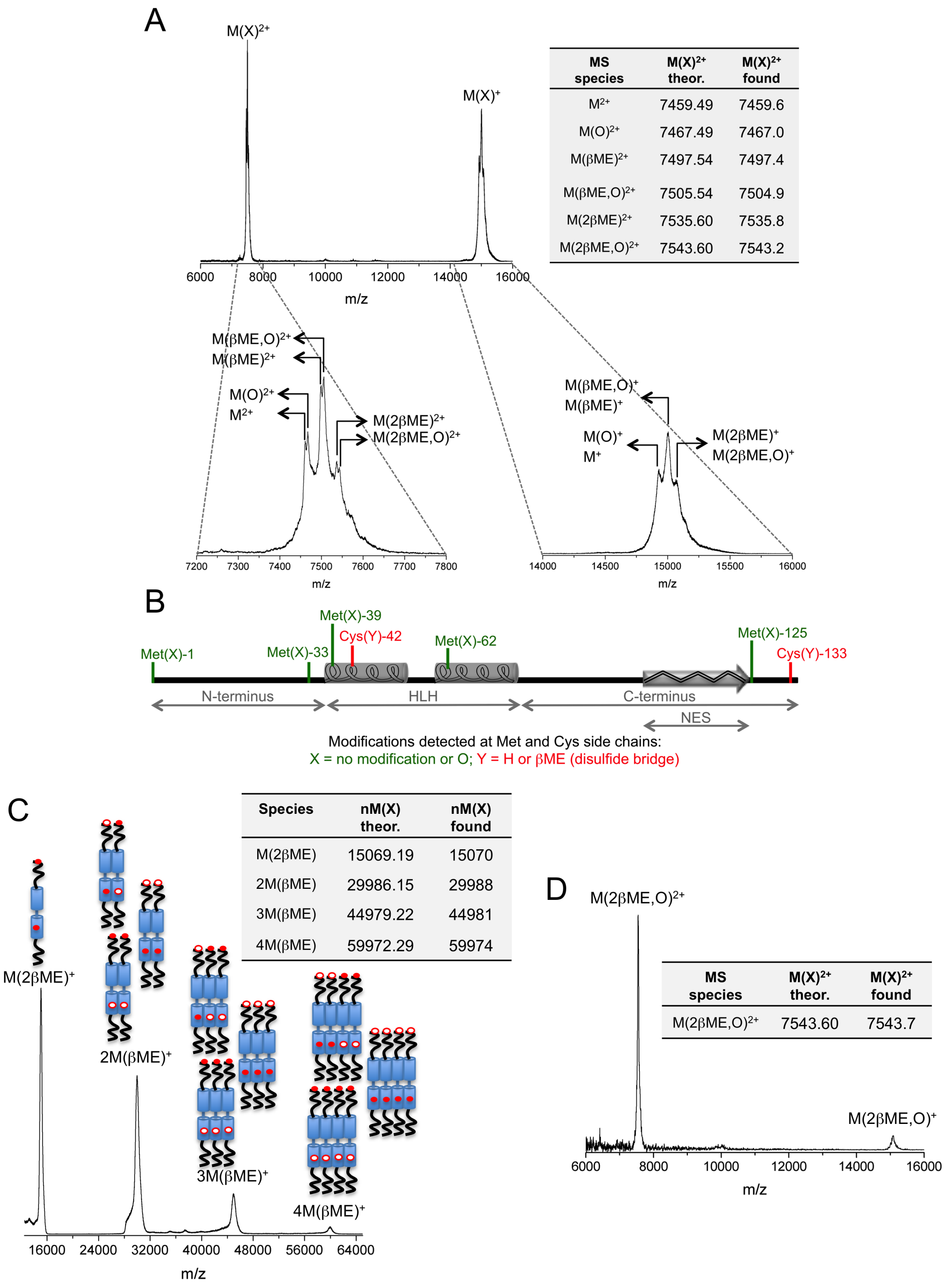

Figure S3. MALDI-TOF-MS of recombinant Id2 from three independent preparations. (A) Three species were identified, based on the $M(X)^{2+}$ peak that was better resolved than the $M(X)^{+}$ peak: the unmodified protein and two oxidized species containing one or two mixed disulfide bridges with βME. Each species coexisted with a variant containing at least one Met(O). The MS spectra were measured in linear mode. The theoretical masses refer to the average molecular weights. (B) Summary of the modifications detected at Met and Cys side chains. HLH = helix-loop-helix; NES = nuclear export signal. (C) Four species were identified: the Id2 monomer containing two mixed disulfide bridges with βME, and the dimer, trimer and tetramer of Id2, in which two, three or four βME groups, respectively, were present. The number of βME groups in the trimers and tetramers excludes the possibility that all Id2 subunits were cross-linked by disulfide bonds. However, the presence of a disulfide bond between two free Cys residues cannot be excluded. In the case of the trimers and tetramers, other combinations of the positions of βME and free Cys, which are represented by red and white circles, respectively, may be possible. The MS spectrum was measured with linear mode. The theoretical masses refer to the average molecular weights. (D) One species was identified: the Id2 monomer containing two mixed disulfide bridges with βME and one Met(O). The MS spectrum was measured with linear mode. The theoretical mass refers to the average molecular weight. This sample was used for further investigation and is referred to as Id2'.

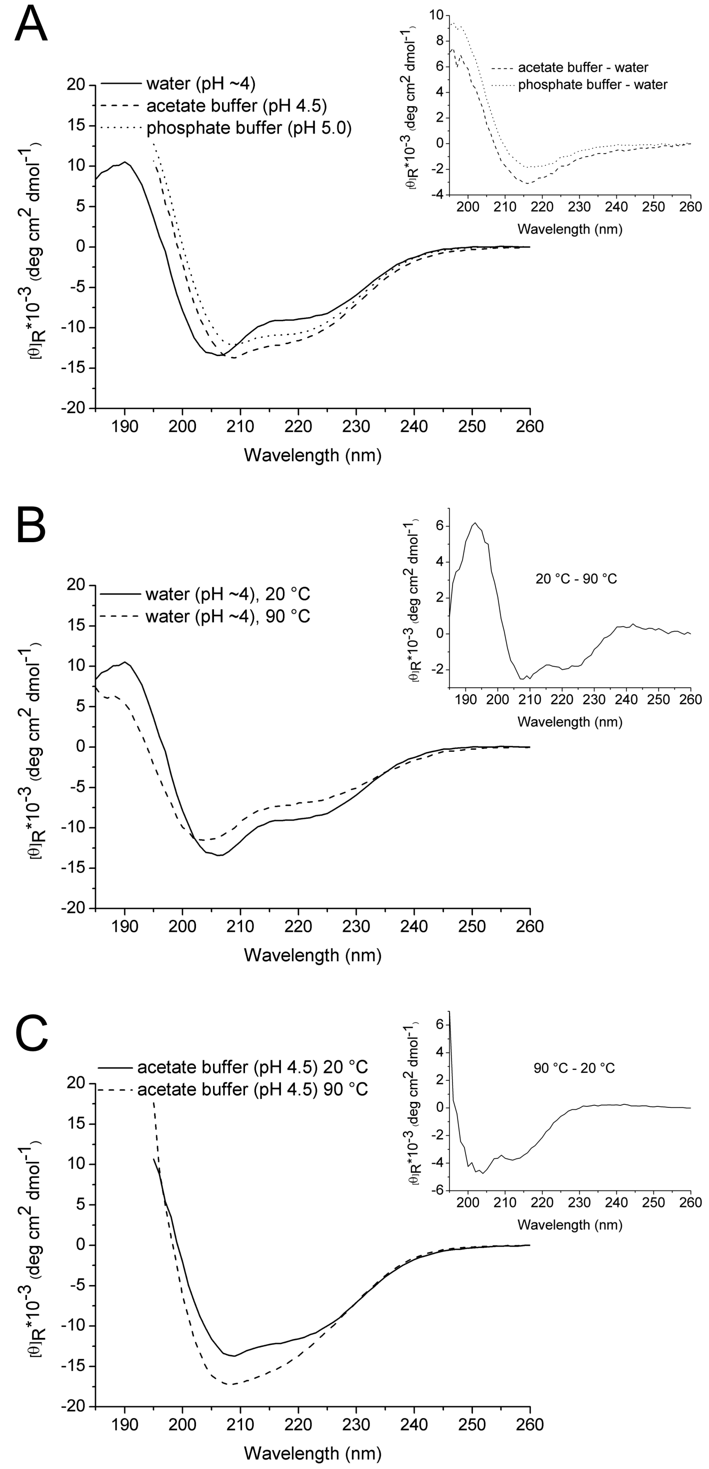

Figure S4. CD spectra of recombinant Id2' (30 μM). (A) CD curves in 100 mM buffer (pH 4.5 or 5.0) or in pure water (pH~4). The inset shows the CD component that appears in buffered solutions (CD difference spectrum between buffer and water solutions), which suggests gain of β-strands. (B) CD curves in pure water at 20 °C and 90 °C. The inset shows the CD component that disappears upon heating (CD difference spectrum between 20 °C and 90 °C), which suggests loss of helical elements. (C) CD curves in sodium acetate buffer at 20 °C and 90 °C. The inset shows the CD component that appears upon heating (CD difference spectrum between 90 °C and 20 °C), which suggests gain of β-strands and aggregates. The CD spectra of the buffered solutions could be recorded only until 195 nm due to exceeding detector voltage below this wavelength. The CD unit is the molar residue ellipticity (MRE = $[\theta]_R$) that was divided by 10^3 for convenience of representation of the Y-axis.

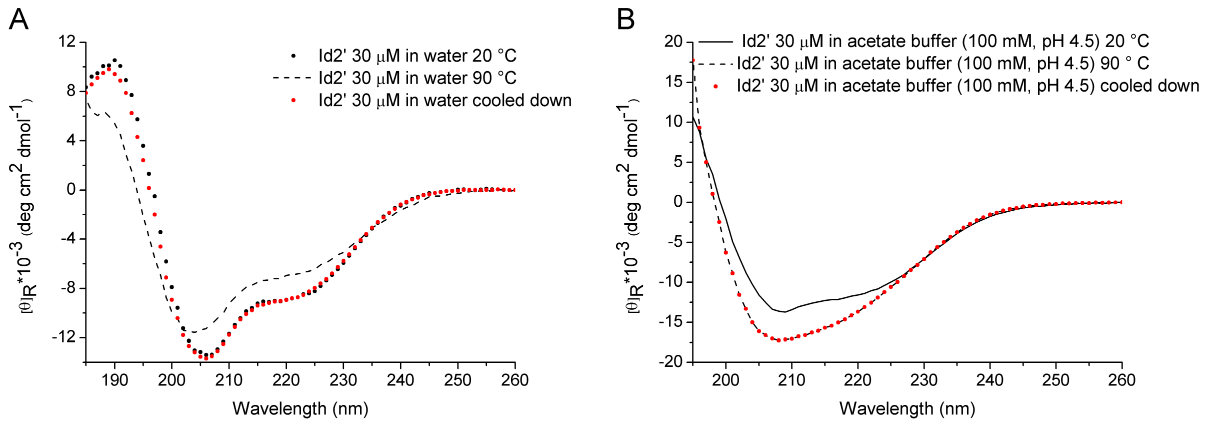

Figure S5. Different reversibility of the secondary-structure changes upon heating/cooling of non-buffered and buffered Id2'. Only the thermal transition of non-buffered Id2' is reversible. (A) Id2' (30 μM) in water (pH~4) at 20 °C and after heating up/cooling down to 90/20 °C. (B) Id2' (30 μM) in sodium acetate buffer (100 mM, pH 4.5) at 20 °C and after heating up/cooling down to 90/20 °C. The CD unit is the molar residue ellipticity (MRE = $[\theta]_R$) that was divided by 10^3 for convenience of representation of the Y-axis.

A

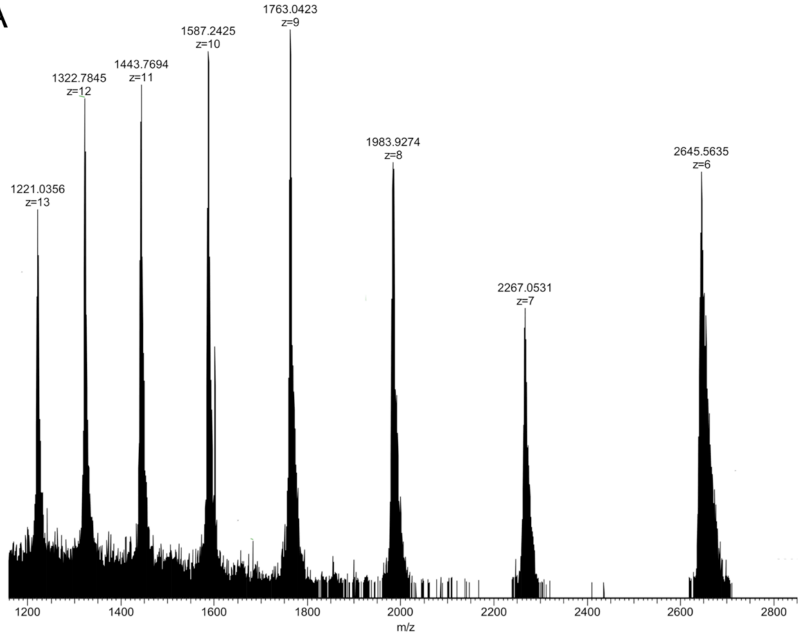

$M(2\beta ME)^{n+}$	$M(2\beta ME)^{n+}$ theor.	$M(2\beta ME)^{n+}$ found
6+	2649.89	2645.56
7+	2271.48	2267.05
8+	1987.67	1983.93
9+	1766.93	1763.04
10+	1590.34	1587.24
11+	1445.85	1443.77
12+	1325.45	1322.78
13+	1223.57	1221.04

$M(2\beta ME)$ theor. for $^{13}C_{650}H_{1062}{}^{15}N_{182}O_{204}S_7({}^{12}C_4H_{10}O_2S_2) = 15893.29$
$M(2\beta ME)$ found = 15863.29
$^{13}C,{}^{15}N$-labeling = ~96%

B

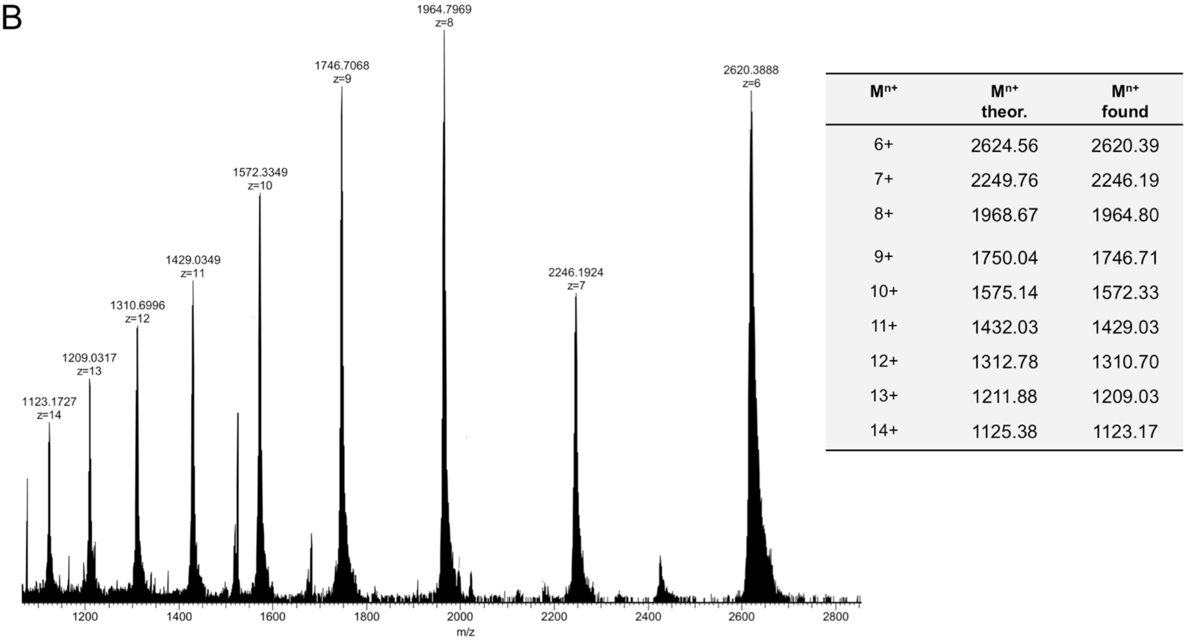

M^{n+}	M^{n+} theor.	M^{n+} found
6+	2624.56	2620.39
7+	2249.76	2246.19
8+	1968.67	1964.80
9+	1750.04	1746.71
10+	1575.14	1572.33
11+	1432.03	1429.03
12+	1312.78	1310.70
13+	1211.88	1209.03
14+	1125.38	1123.17

M theor. for $^{13}C_{650}H_{1064}{}^{15}N_{182}O_{204}S_7 = 15741.29$
M found = 15711.93
$^{13}C,{}^{15}N$-labeling = ~96%

Figure S6. ESI-MS of the $^{13}C,{}^{15}N$-labeled recombinant Id2 protein used for the NMR experiments and referred to as Id2". (A) MS of the protein containing two mixed disulfide bridges with βME. (B) The MS sample in (A) was treated with 5 mM TCEP for 15 min at 60 °C to reduce the mixed disulfide bonds with βME. Both measurements indicate a ~96% degree of isotope labeling. The theoretical masses refer to the mono-isotopic molecular masses.

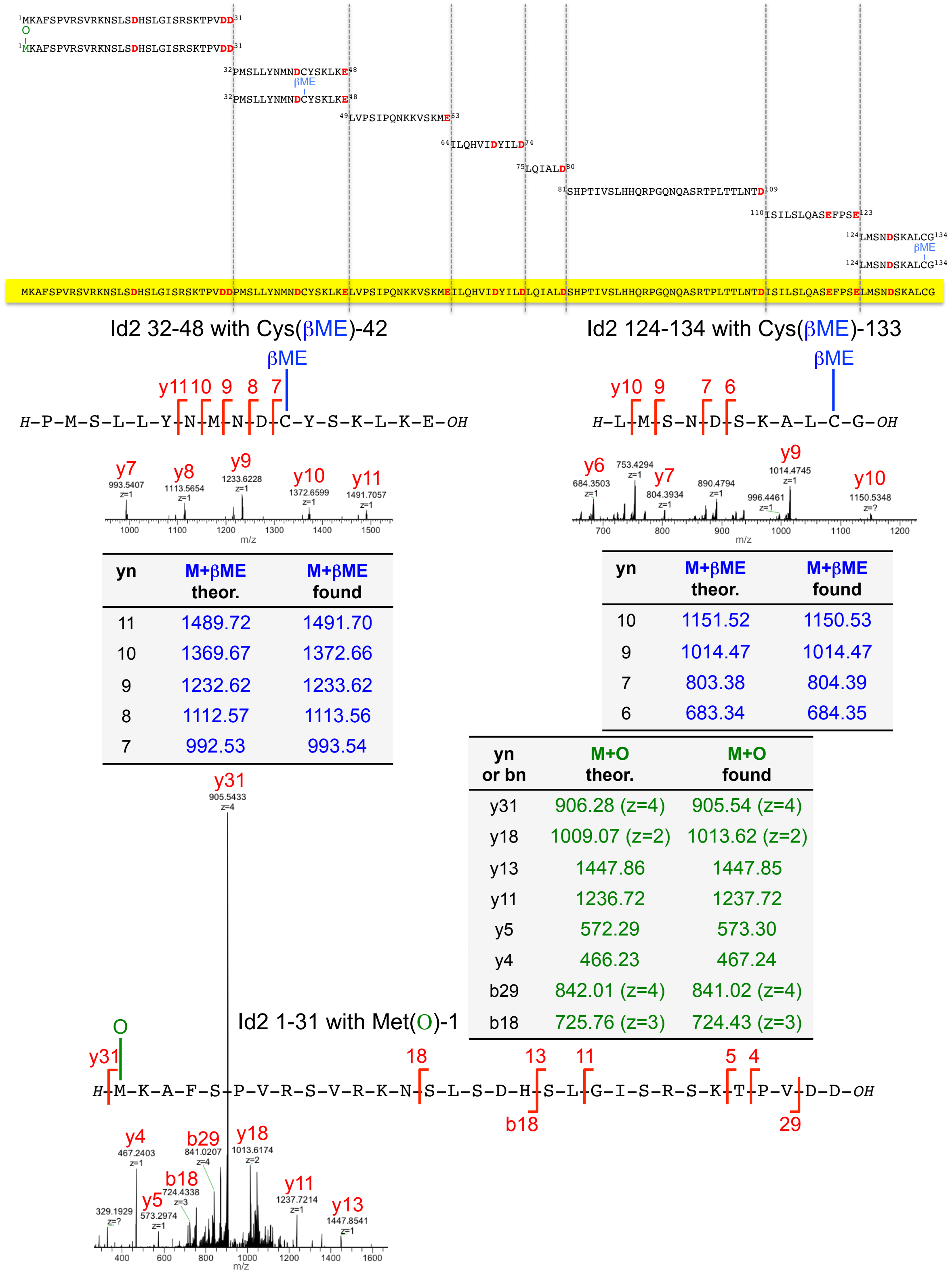

yn	M+βME theor.	M+βME found
11	1489.72	1491.70
10	1369.67	1372.66
9	1232.62	1233.62
8	1112.57	1113.56
7	992.53	993.54

yn	M+βME theor.	M+βME found
10	1151.52	1150.53
9	1014.47	1014.47
7	803.38	804.39
6	683.34	684.35

yn or bn	M+O theor.	M+O found
y31	906.28 (z=4)	905.54 (z=4)
y18	1009.07 (z=2)	1013.62 (z=2)
y13	1447.86	1447.85
y11	1236.72	1237.72
y5	572.29	573.30
y4	466.23	467.24
b29	842.01 (z=4)	841.02 (z=4)
b18	725.76 (z=3)	724.43 (z=3)

Figure S7. Fragments of the ^{13}C,^{15}N-labeled recombinant Id2 protein (used for the NMR experiments and referred to as Id2") upon digestion with Glu-C and treatment with 5 mM TCEP. The detection was performed by LC-ESI-MS/MS. For the N-terminal fragment 1-31, both variants with reduced and oxidized Met-1 were detected. For the two fragments containing Cys-42 or Cys-133, both variants with and without the mixed disulfide with βME were detected, indicating that the TCEP treatment did not completely reduce the disulfide bonds. The MS/MS data for the oxidized forms of fragments 1-31, 32-48 and 124-134 are shown.

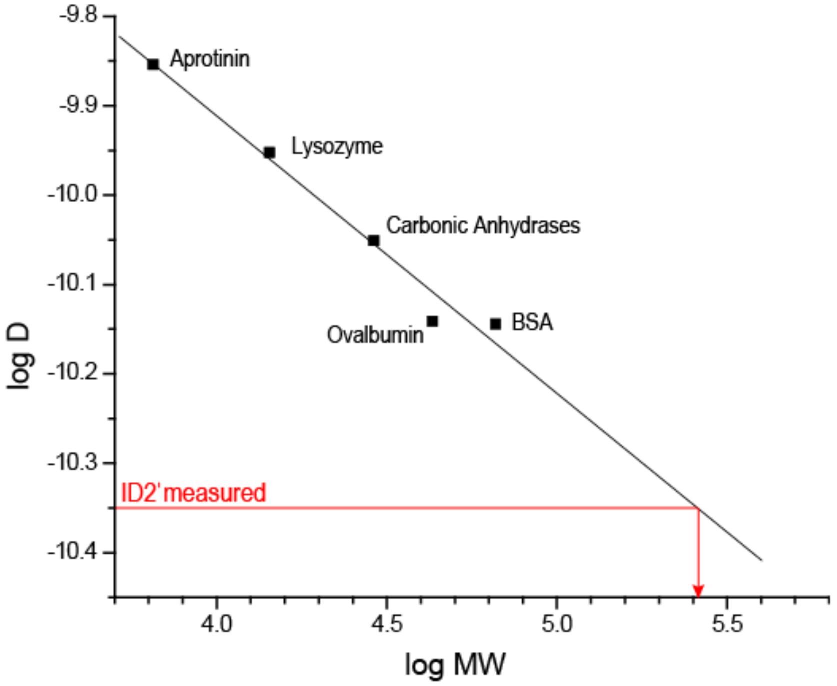

Figure S8. Result of a 2D DOSY experiment with unlabeled Id2' (0.27 mM) in 94% H_2O/6% D_2O at 298 K together with a calibration curve obtained from different protein standards measured under comparable conditions.

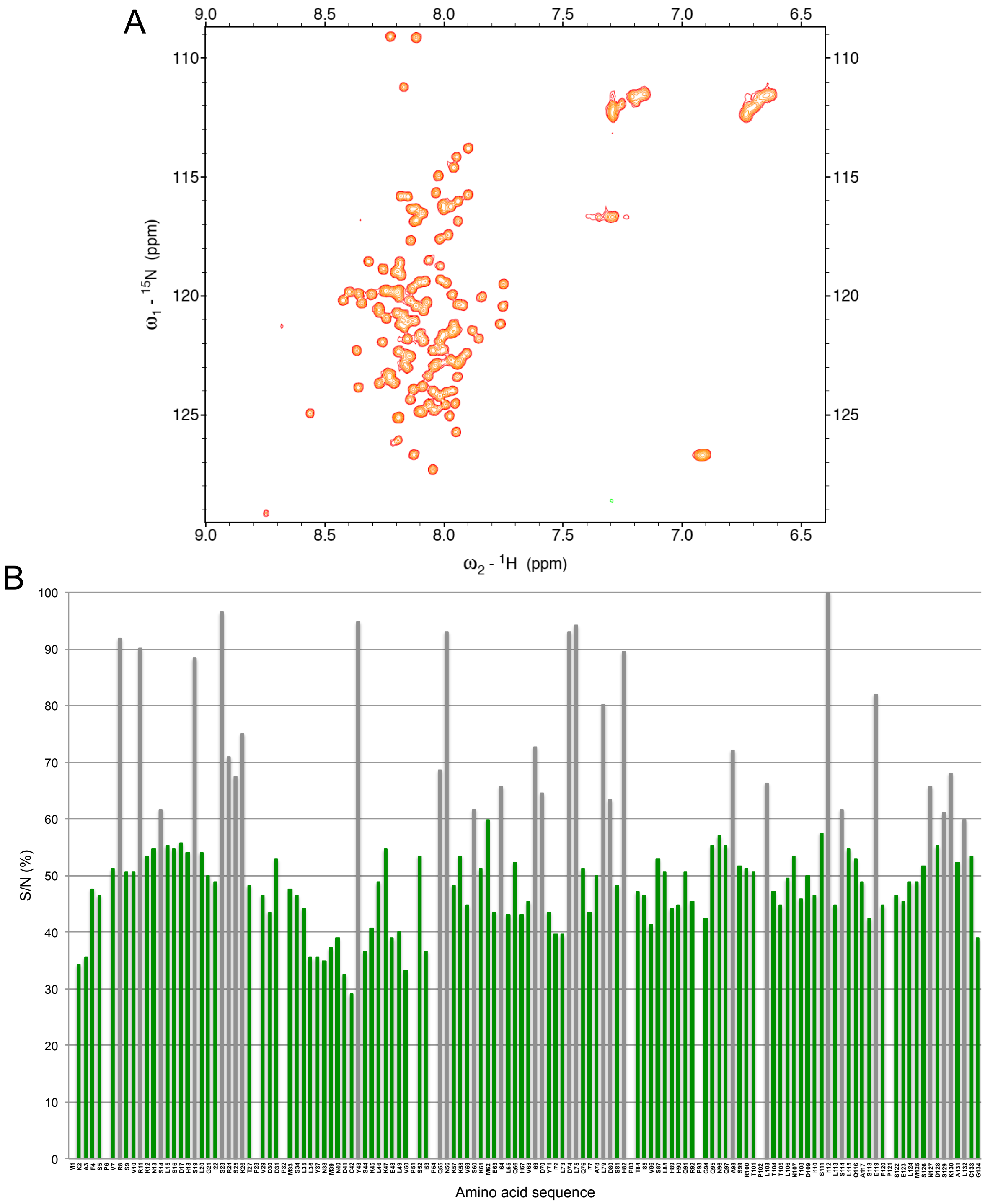

Figure S9. HSQC spectrum of ^{13}C,^{15}N-Id2" (0.74 mM) in 8 M urea with 5% D_2O (pH 2.3, adjusted with HCl until reaching a final chloride concentration of 180 mM). (A) The spectrum was measured at 298 K. (B) The S/N ratio of the ^{15}N-H crosspeaks normalized to 100% is shown. The bars in gray correspond to partially overlapped crosspeaks.

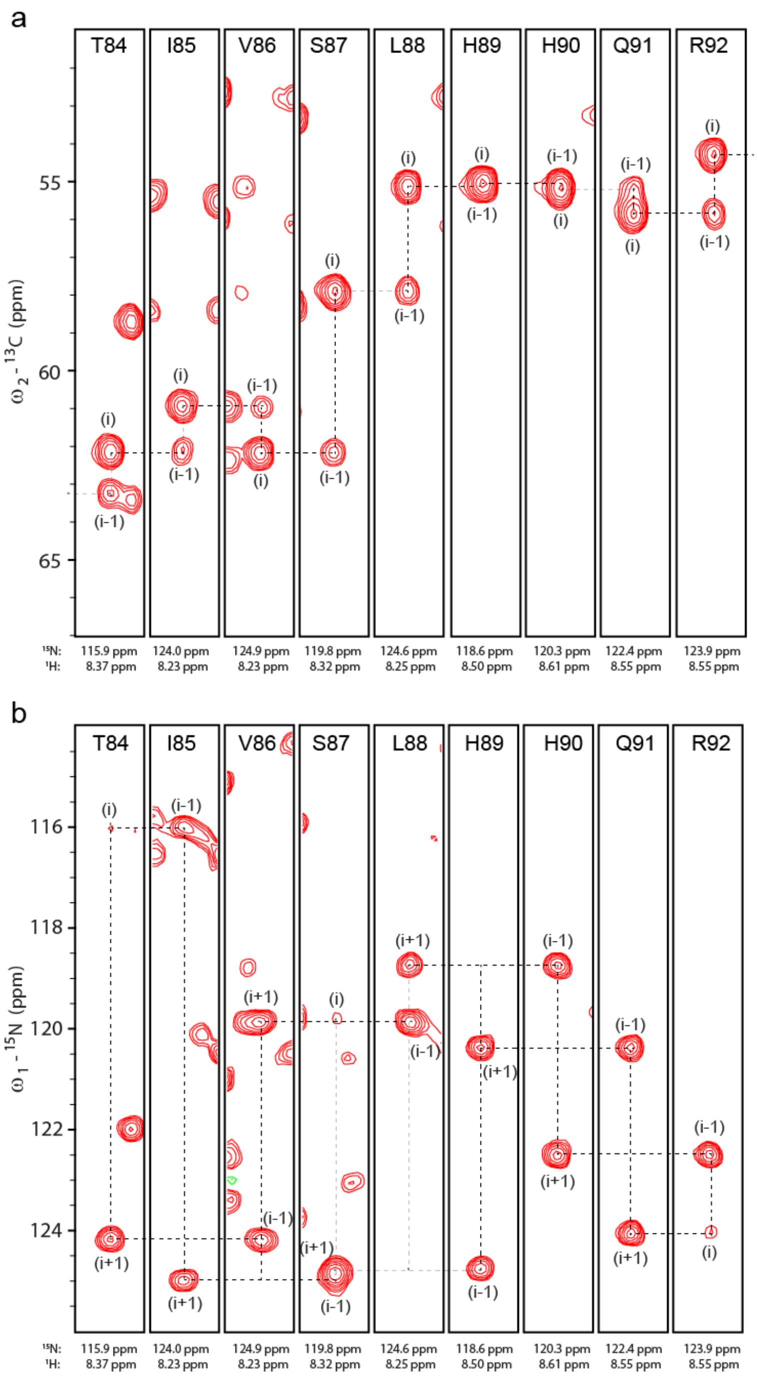

Figure S10. Sequential backbone assignment of ^{13}C,^{15}N-Id2" (0.74 mM) in 8 M urea with 5% D_2O (pH 2.3, adjusted with HCl until reaching a final chloride concentration of 180 mM) by 3D (H)NCANH spectrum. (A) Sequential walk using the 3D HNCA spectrum recorded with 8 transients and 2048x96x56 data points. Shown are strips of residues Thr-84 to Arg-92, which are flanked by proline residues. The low dispersion in ^{13}C is especially difficult for consecutive amino acids of the same type. (B) Corresponding strips of a 3D (H)NCANH spectrum recorded with 48 transients and 2048x56x56 data points.

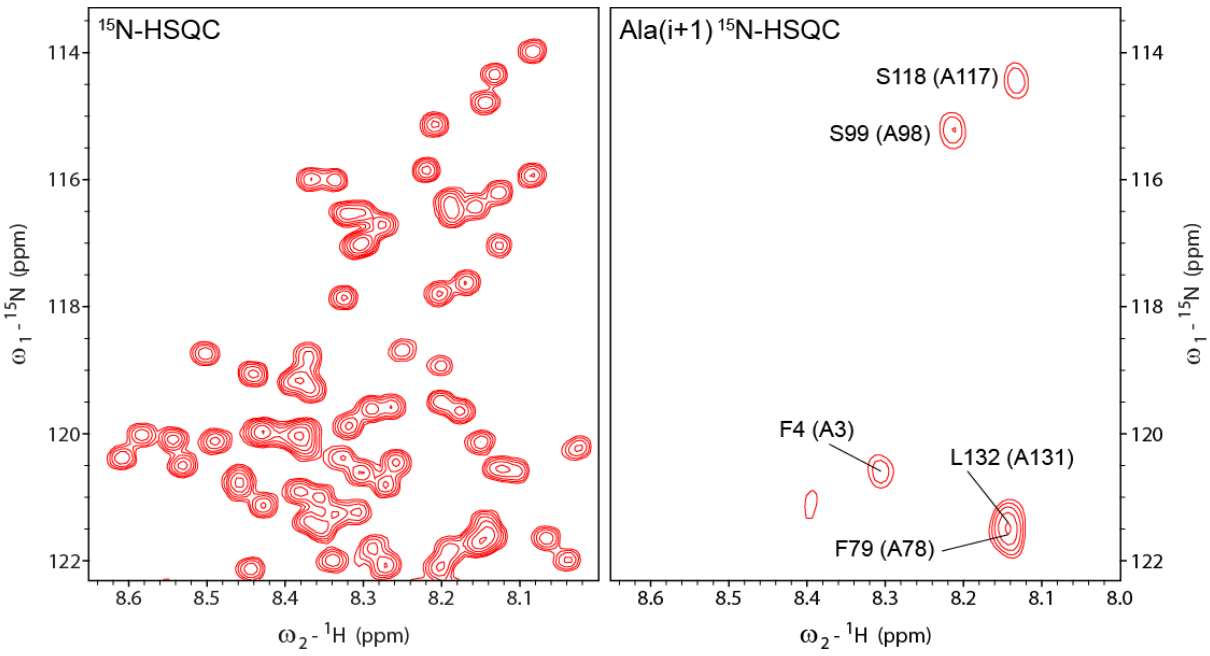

Figure S11. Sequential backbone assignment of $^{13}C,^{15}N$-Id2" (0.74 mM) in 8 M urea with 5% D_2O (pH 2.3, adjusted with HCl until reaching a final chloride concentration of 180 mM) supported by amino acid-type selective spectra. A portion of a conventional ^{15}N-HSQC spectrum is shown on the left. An alanine selective ^{15}N-HSQC, recorded with 48 transients, displays ^{15}N-H correlations of residues following alanine (shown on the right). The Id2 protein sequence contains five alanine residues and all five (i+1) neighbors show up in the spectrum.

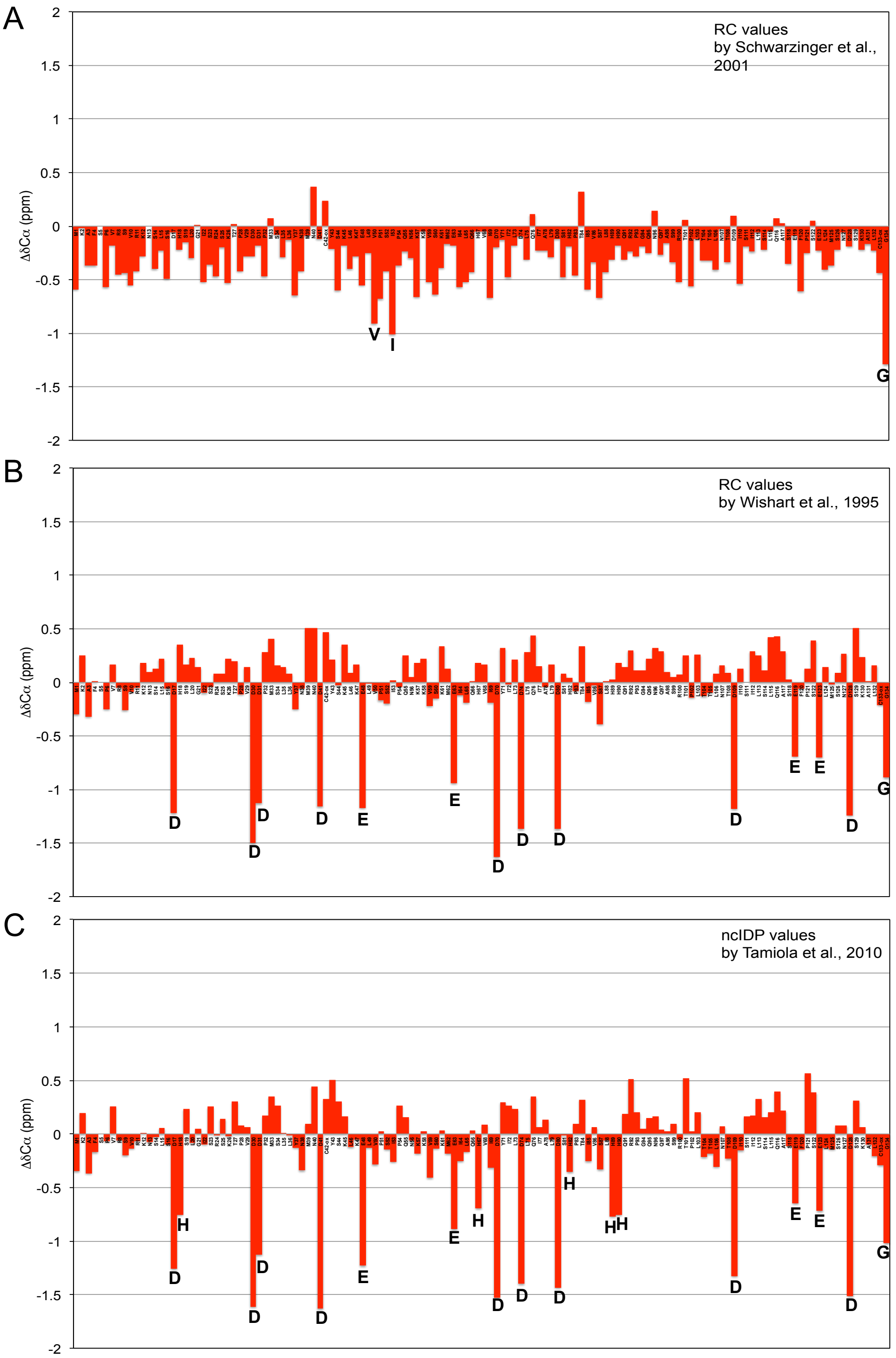

Figure S12. Comparison of the $^{13}C\alpha$ chemical shifts of $^{13}C,^{15}N$-Id2'' (0.74 mM) in 8 M urea (pH 2.3) with known reference random coil values. The reference random coil (RC) values were from (A) Schwarzinger et al. (obtained from GGXGG in 8 M urea at pH 2.3 and neighbor corrected for $^{13}C\alpha$) [1, 2], (B) Wishart et al. (obtained from GGXAGG or GGXPGG in 1 M urea at pH ~5) [3], (C) Tamiola et al. (obtained from IDPs and neighbor corrected, except for Cys-ox that were from Wishart et al. [3]) [4]. The Id2'' RC shifts were in general in good agreement with all three libraries, confirming the denatured state of the Id2'' protein under these conditions. However, following exceptions could be found: the acidic residues Asp and Glu of Id2'' were significantly up-field shifted in comparison to those shown by Wishart's peptide models measured at pH ~5 as well as by the IDPs from the ncIDP library (panels B and C). Such discrepancy for the acidic residues was expected, as they are differently ionized at pH 2.3 and pH ~5. Interestingly, an up-field shift of the His RC

values of Id2" could be also noticed in comparison to the RC shifts of His from the ncIDP library (panel C). This suggests that the His residues are mainly uncharged in the ncIDP library of proteins under native conditions. Comparison with the Schwarzinger library was not expected to show exceptions; however, we found an up-field shift of the ^{13}Cα of Val-50 and Ile-53, both residues being followed by a proline residue.

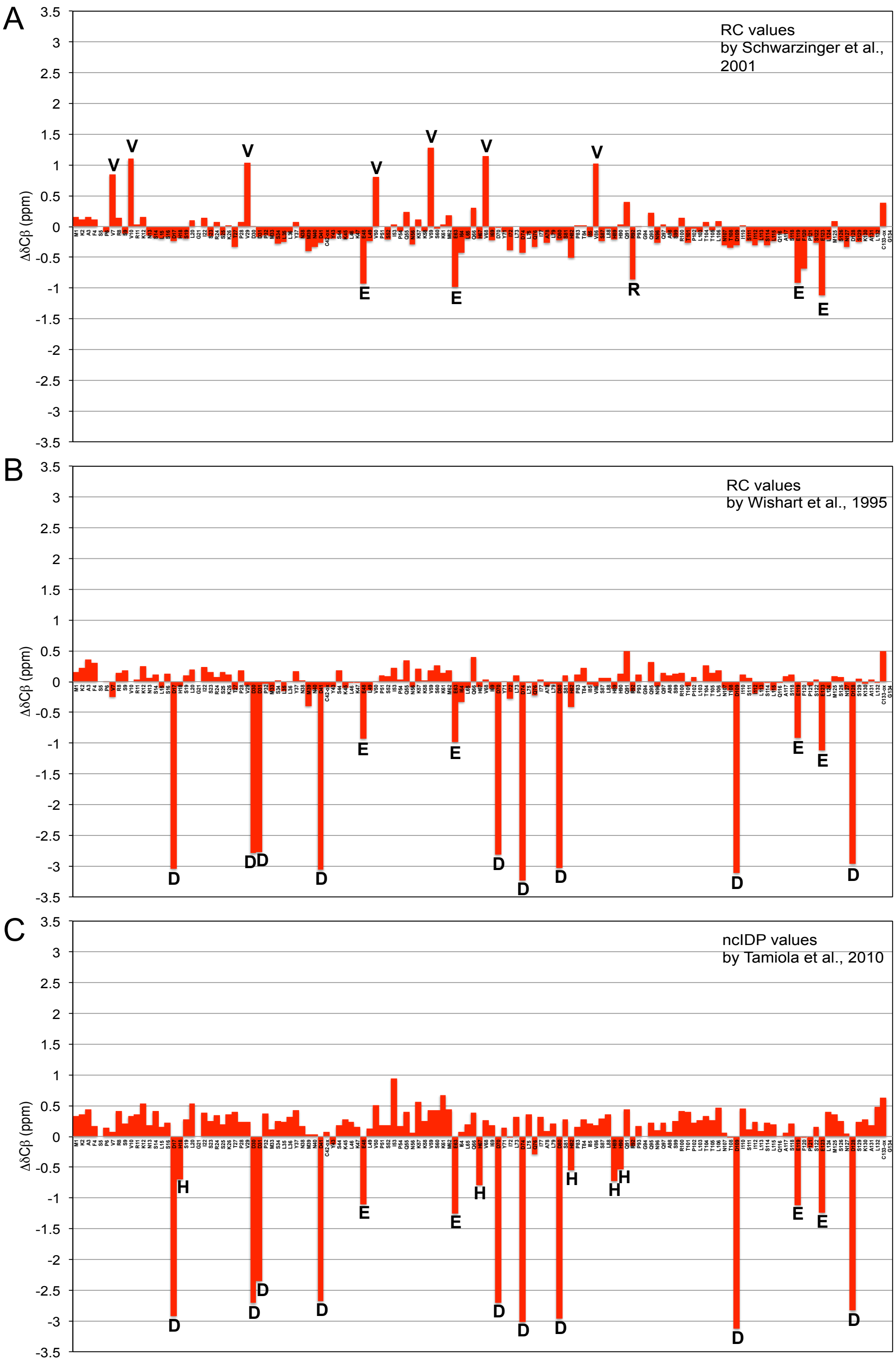

Figure S13. Comparison of the ^{13}Cβ chemical shifts of ^{13}C,^{15}N-Id2" (0.74 mM) in 8 M urea (pH 2.3) with known reference random coil values. The reference random coil (RC) values were from (A) Schwarzinger et al. (obtained from GGXGG in 8 M urea at pH 2.3) [1, 2], (B) Wishart et al. (obtained from GGXAGG or GGXPGG in 1 M urea at pH ~5) [3], (C) Tamiola et al. (obtained from IDPs and neighbor corrected, except for Cys-ox that were from Wishart et al. [3]) [4]. The Id2" RC shifts were in general in good agreement with all three libraries, confirming the denatured state of the Id2" protein under these conditions. However, following exceptions could be found: the acidic residues Asp and Glu of Id2" were significantly up-field shifted in comparison to those shown by Wishart’s peptide models measured at pH ~5 as well as by the IDPs from the ncIDP library (panels B and C). Such discrepancy for the acidic residues was expected, as they are differently ionized at pH 2.3 and pH ~5. Interestingly, an up-field shift of the His RC values of Id2" could be also noticed

in comparison to the RC shifts of His from the ncIDP library (panel C). This suggests that the His residues are mainly uncharged in the ncIDP library of proteins under native conditions. Comparison with the Schwarzinger library was not expected to show exceptions; however, the ^{13}Cβ of all seven valine residues of Id2" and all four glutamic acid residues were found to be down-field and up-field shifted, respectively.

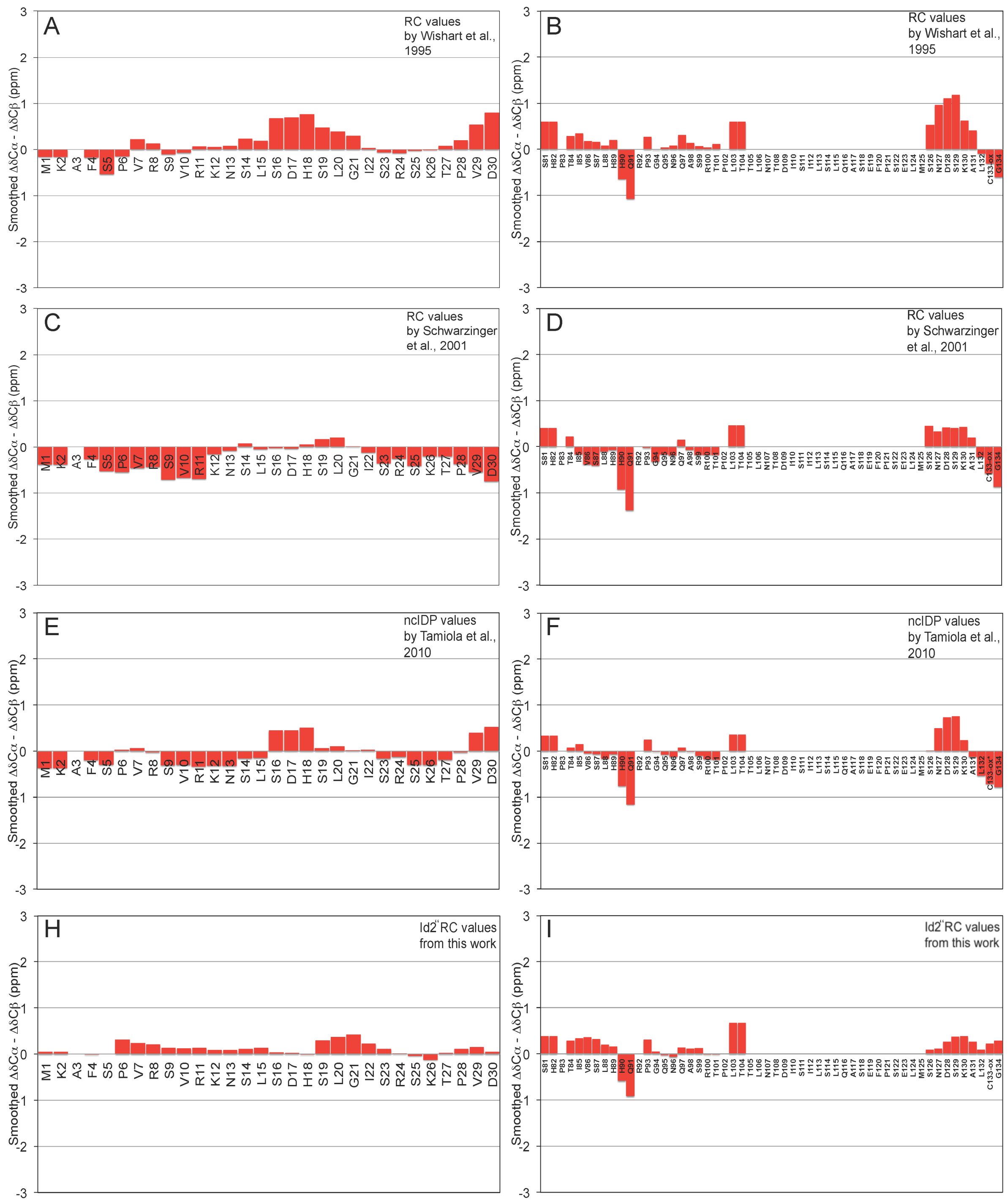

Figure S14. Deviations of ^{13}Cα and ^{13}Cβ chemical shifts from the RC values (secondary chemical shifts) for the N-terminal (left) and C-terminal (right) flexible regions of ^{13}C,^{15}N-Id2" (0.86 mM) in water. The reference random coil (RC) values were from (A and B) Wishart et al. (obtained from GGXAGG or GGXPGG in 1 M urea at pH ~5) [3], (C and D) Schwarzinger et al. (obtained from GGXGG in 8 M urea at pH 2.3 and neighbor corrected for ^{13}Cα) [1, 2], (E and F) Tamiola et al. (obtained from IDPs and neighbor corrected, except for Cys-ox that were from Wishart et al. [3]) [4], and (H and G) this work (obtained from the Id2" sample in 8 M urea at pH 2.3). The secondary chemical shifts were smoothed by a three-point function [5].

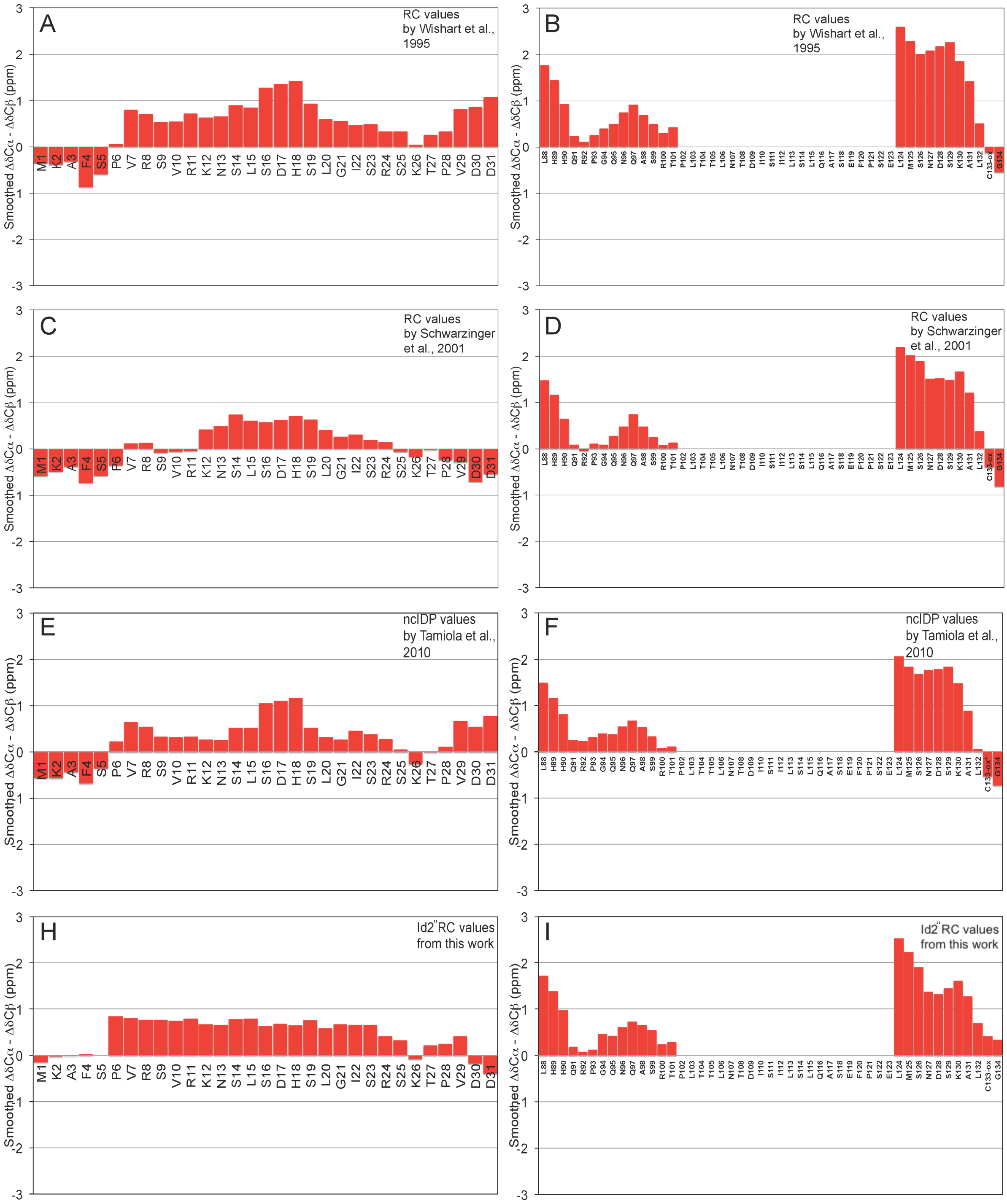

Figure S15. Deviations of ^{13}Cα and ^{13}Cβ chemical shifts from the RC values (secondary chemical shifts) for the N-terminal (left) and C-terminal (right) flexible regions of ^{13}C,^{15}N-Id2" (0.5 mM) in water/TFE (70:30, v/v). The reference random coil (RC) values were from (A and B) Wishart et al. (obtained from GGXAGG or GGXPGG in 1 M urea at pH ~5) [3], (C and D) Schwarzinger et al. (obtained from GGXGG in 8 M urea at pH 2.3 and neighbor corrected) [1, 2], (E and F) Tamiola et al. (obtained from IDPs and neighbor corrected, except for Cys-ox that were from Wishart et al. [3]) [4], and (H and G) this work (obtained from the Id2" sample in 8 M urea at pH 2.3). The secondary chemical shifts were smoothed by a three-point function [5].

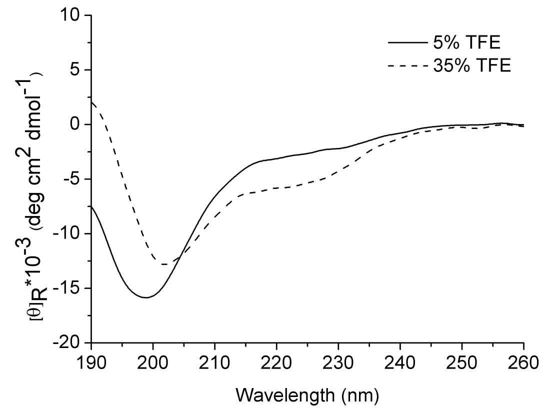

Figure S16. CD spectra of a synthetic peptide reproducing the N-terminal region of Id2 (residues 1-35), dissolved in 100 mM phosphate buffer (pH 7) at the concentration of 70 μM, and containing 5% or 35% TFE. The peptide was synthesized as described previously [6].

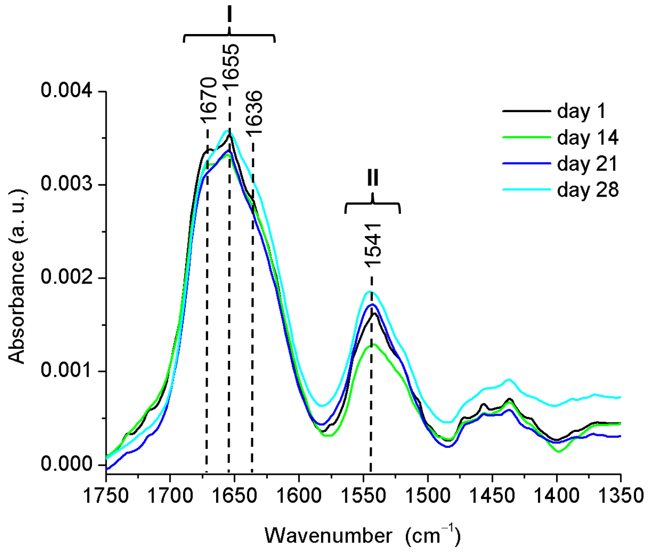

Figure S17. ATR-FTIR spectra of Id2' in H_2O (1 mM) upon aging.

Table S1. Buffer screening to determine the pH-dependent solubility of recombinant Id2'.

**Buffer**	**0 min 37 °C**[a]	**15 min 37 °C**[a]	**24 h 37 °C**[a]	**24 h at 37 °C + 5 d at 21 °C**[a]
100 mM potassium phosphate pH 5.0	0	0	0	0
100 mM potassium phosphate pH 6.0	0	1	2	2
100 mM potassium phosphate pH 7.0	1	3	4	5
100 mM potassium phosphate pH 7.4	1	3	4	5
100 mM sodium phosphate pH 5.5	0	0	0	0
100 mM sodium phosphate pH 6.5	1	3	4	2
100 mM sodium phosphate pH 7.5	2	3	4	5
100 mM sodium acetate pH 4.5	0	0	0	0
100 mM sodium citrate pH 4.7	0	1	2	1
100 mM sodium acetate pH 5.0	0	0	0	0
100 mM sodium citrate pH 5.5	1	3	4	5
100 mM HEPES pH 7.0 + 20 mM Ca^{2+}	2	3	4	5
100 mM ammonium acetate pH 7.3	2	3	4	5
50 mM potassium phosphate pH 8.0 + 300 mM NaCl	1	3	4	5
50 mM potassium phosphate pH 5.7 + 100 mM NaCl	0	0	0	0
100 mM MES pH 5.8	0	0	0	0
100 mM MES pH 6.2	0	0	0	0
100 mM MES pH 6.5	0	1	1	1
100 mM HEPES pH 7.0	2	3	4	5
100 mM HEPES pH 8.0	2	3	4	5
100 mM TRIS pH 7.5	2	3	4	5
100 mM TRIS pH 8.0	2	3	4	5
100 mM TRIS pH 8.5	2	3	4	5

[a]Precipitation score from 0 to 5, with 0 for no precipitation and 5 for precipitate completely covering the drop.

Table S2. Chemical shifts assignment (ppm) of $^{13}C,^{15}N$-Id2" (0.86 mM) in H_2O/D_2O (93:7, v/v), measured at 313 K, referenced to DSS. The asterisk is given to residues that could not be assigned unequivocally.

Residue	^{13}CO	^{13}Cα	^{13}Cβ	^{15}N	^{15}NH
M1	172.0	55.1	33.0		
K2	175.3	56.5	33.2	124.6	8.58
A3	177.0			126.1	8.27
F4	175.3	57.7	39.9	120.4	8.20
S5	184.2	55.8	63.9	119.6	8.06
P6	176.7	63.2	32.1		
V7	176.2	62.6	32.5	120.4	8.07
R8	176.0	56.0	31.0	125.0	8.30
S9	174.3	58.1	63.9	117.4	8.20
V10	175.9	62.3	32.8	122.0	8.12
R11	176.1	56.0	30.9	125.0	8.29
K12	176.2	56.4	33.2	123.4	8.32
N13	175.2	53.3	39.0	120.2	8.42
S14	174.5	58.5	63.9	116.4	8.24
L15	177.6	55.6	42.3	123.8	8.20
S16	174.4	58.3	63.9	115.7	8.13
D17	175.4	53.3	38.6	120.9	8.23
H18	174.4	55.4	28.8	118.9	8.37
S19	174.5	58.6	63.9	116.7	8.23
L20	177.8	56.0	42.6	123.9	8.22
G21	174.1	45.4		109.1	8.26
I22	176.4	61.1	39.0	119.6	7.85
S23	174.6	58.3	63.9	119.7	8.31
R24	176.2	56.1	30.9	123.4	8.31
S25	174.4	58.4	64.1	116.7	8.20
K26*	176.4	56.3	33.2	123.3	8.32
T27*	172.8	60.0	69.8	117.8	8.08
P28	176.7	63.3	32.1		
V29	175.7	62.4	32.7	119.7	8.07
D30	174.2	52.8	38.6	121.8	8.34
D31				121.1	8.11
...					
S81*		58.8	63.7		
H82*		53.6	28.6	119.7	8.16
P83	177.1				
T84	174.6	62.2	70.0	115.0	8.24
I85	176.2	61.3	38.7	123.3	8.11
V86	176.0	62.5	32.8	123.6	8.06
S87	176.4	58.1	63.9	118.9	8.17
L88	177.1	55.5	42.4	123.9	8.11
H89	174.1	55.2	29.0	117.6	8.32
H90	175.5	56.0	29.9	121.8	8.40
Q91	174.2	54.2	30.1	123.5	8.40

R92					
P93	177.4	63.5	32.0		
G94	174.2	45.4		109.3	8.42
Q95	175.8	56.0	30.3	119.5	8.09
N96	175.2	53.5	38.8	119.7	8.46
Q97	175.7	56.2	29.5	120.7	8.30
A98	177.7	52.8	19.3	124.7	8.24
S99	174.5	58.4	63.9	114.6	8.11
R100	176.1	56.2	30.9	122.6	8.24
T101	172.9	60.0	69.9	117.0	8.08
P102					
L103		55.8	42.4		
T104		62.3	69.8	113.8	7.98
T105*				116.0	7.97
...					
S126	176.1	58.7	63.7		
N127	175.2	53.5	38.8	120.1	8.33
D128		53.4	38.4	118.9	8.29
S129	175.4	59.2	63.8	115.9	8.12
K130	176.5	56.7	32.8	122.4	8.11
A131	177.7	52.8	19.3	123.6	7.98
L132	177.3	55.5	42.5	120.1	7.97
C133-ox	174.7	55.4	41.8	118.0	8.17
G134		44.6		111.4	8.16

Table S3. Chemical shifts assignment (ppm) of $^{13}C,^{15}N$-Id2" (0.5 mM) in TFE/H_2O/D_2O (30:63:7, v/v), measured at 298 K, referenced to DSS. The asterisk is given to residues that could not be assigned unequivocally.

Residue	^{13}CO	$^{13}C\alpha$	$^{13}C\beta$	^{15}N	^{15}NH
M1		55.1	33.3		
K2		56.4	33.4	124.1	8.63
A3		52.3	19.3	125.2	8.28
F4		57.6	40.0	119.5	8.09
S5		55.5	64.1	118.2	7.98
P6		63.5	32.0		
V7		63.2	32.5	118.5	7.83
R8	176.3	56.4	30.7	123.0	8.17
S9	174.5	58.5	63.9	115.6	8.17
V10	176.3	62.9	32.7	121.3	8.05
R11	176.4	56.5	30.8	123.6	8.27
K12	176.5	56.9	33.2	122.2	8.33
N13	175.5	53.7	38.8	119.1	8.43
S14	174.9	59.2	64.0	115.9	8.30
L15	177.9	56.2	42.1	123.0	8.22
S16	174.7	59.0	63.9	114.5	8.12
D17	175.9	54.0	38.9	120.5	8.24
H18	174.6	56.0	28.4	118.3	8.39
S19	174.7	59.2	64.0	115.7	8.28
L20	178.0	55.9	42.6	123.1	8.18
G21	174.4	45.7		108.1	8.28
I22	176.5	61.7	38.9	119.1	7.81
S23	174.8	58.6	63.9	118.3	8.25
R24	176.3	56.5	30.7	122.5	8.30
S25	174.5	58.6	64.0	115.7	8.12
K26	176.1	56.4	33.1	122.6	8.29
T27	173.1	59.7	69.9	115.7	8.05
P28	176.7	63.7	31.7		
V29	175.6	62.5	32.7	117.4	7.85
D30	174.2	53.1	38.7	120.7	8.24
D31	174.1	51.3	39.1	118.5	8.09
...					
L88*	178.1	56.6	42.6		
H89*	174.3	56.4	28.3	115.0	8.03
H90*	174.0	55.8	29.0	117.8	8.18
Q91*	175.4	56.0	29.9	120.4	8.23
R92*	174.1	54.1	30.2	122.5	8.41
P93	177.3	63.6	31.9		
G94	174.4	45.3	0.0	108.9	8.49
Q95	175.9	56.3	29.5	119.3	8.16
N96	175.4	53.6	38.7	119.1	8.54

Q97	176.0	56.6	29.3	120.5	8.42
A98	177.8	53.1	18.7	123.8	8.30
S99	174.4	58.6	64.0	113.6	8.08
R100	175.8	56.3	30.7	122.1	8.20
T101	173.7	60.2	69.8	115.1	8.16
P102					
L103					
T104*				112.5	7.93
T105*				113.2	8.12
...					
E119*				119.6	7.89
F120*				120.1	8.04
...					
L124	178.6	56.8	41.5		
M125	177.4	56.9	31.9	116.8	7.97
S126	175.2	59.9	63.7	114.4	7.99
N127	175.8	54.5	38.5	119.6	8.23
D128	176.1	54.0	38.1	118.6	8.42
S129	175.4	60.2	63.6	115.5	8.19
K130	177.3	57.5	32.4	121.8	8.07
A131	178.2	53.5	18.6	122.1	7.93
L132	177.5	55.8	42.4	118.0	7.86
C133-ox	175.0	55.4	41.8	116.4	8.09
G134		44.7	0.0	110.9	8.06

Table S4. Chemical shifts assignment (ppm) of ^{13}C,^{15}N-Id2" (0.74 mM) in 8 M urea (pH 2.3), measured at 298 K, referenced to DSS (data deposited in the BMRB with accession no. 27358).

Residue	^{13}CO	$^{13}C\alpha$	$^{13}C\beta$	^{15}N	^{15}NH
M1		55.1	33.1		
K2		56.5	33.3	125.1	8.75
A3		52.2	19.5	126.3	8.38
F4		57.7	39.9	120.6	8.30
S5				119.5	8.20
P6	176.6	63.1	32.1		
V7	176.2	62.4	32.7	120.6	8.13
R8	176.1	56.0	31.0	125.3	8.38
S9	174.3	58.0	64.0	117.9	8.33
V10	175.9	62.1	32.9	122.1	8.20
R11	176.1	56.0	30.9	125.3	8.38
K12	176.2	56.4	33.4	123.9	8.46
N13	175.3	53.2	39.0	120.5	8.53
S14	174.6	58.4	63.9	116.5	8.32
L15	177.4	55.3	42.3	124.0	8.28
S16	174.4	58.2	63.9	115.9	8.22
D17	175.0	53.0	38.1	120.9	8.38
H18	174.3	55.4	28.9	119.1	8.44
S19	174.5	58.5	63.9	117.0	8.30
L20	177.7	55.3	42.6	124.1	8.32
G21	173.8	45.2		109.3	8.30
I22	176.3	61.0	39.0	119.7	7.94
S23	174.5	58.3	64.0	120.1	8.38
R24	176.3	56.1	31.0	123.7	8.42
S25	174.5	58.4	64.0	117.0	8.31
K26	176.4	56.4	33.2	123.6	8.41
T27	172.8	60.0	69.7	117.6	8.17
P28	176.6	63.2	32.3		
V29	175.8	62.3	32.8	120.1	8.15
D30	174.2	52.7	38.3	122.1	8.45
D31	172.8	51.1	38.1	120.0	8.40
P32	176.9	63.6	32.1		
M33	176.4	55.8	32.7	119.6	8.27
S34	174.5	58.5	63.8	116.4	8.16
L35	176.9	55.2	42.3	124.2	8.15
L36	176.9	55.2	42.4	122.0	8.04
Y37	175.4	57.7	39.0	120.2	8.03
N38	175.3	53.0	38.9	120.4	8.33
M39	176.1	55.9	32.5	121.2	8.31
N40	175.1	53.6	38.8	118.8	8.37
D41	175.0	53.0	38.0	118.7	8.25
C42-ox	174.4	55.9	41.0	119.0	8.20

Y43	175.7	58.1	38.7	121.7	8.15
S44	174.4	58.3	64.0	117.0	8.13
K45	176.4	56.6	33.0	123.6	8.25
L46	177.2	55.2	42.4	122.9	8.11
K47	176.3	56.4	33.1	122.7	8.34
E48	175.6	55.4	29.0	121.8	8.29
L49	176.8	55.1	42.3	124.5	8.33
V50	174.4	59.7	32.6	122.5	8.19
P51	176.6	63.1	32.2		
S52	174.2	58.1	63.9	116.5	8.30
I53	174.5	58.7	38.9	123.6	8.13
P54	176.8	63.3	32.1		
Q55	175.9	56.0	29.7	120.9	8.46
N56	175.1	53.2	38.8	120.0	8.43
K57	176.3	56.4	33.3	122.1	8.27
K58	176.5	56.4	33.1	123.2	8.34
V59	176.1	62.0	33.1	121.9	8.19
S60	174.5	58.2	64.1	120.2	8.38
K61	176.4	56.5	33.2	123.9	8.39
M62	176.0	55.5	33.1	121.5	8.35
E63	175.6	55.7	28.9	122.5	8.38
I64	176.0	61.0	38.5	123.2	8.23
L65	177.0	54.9	42.3	126.9	8.31
Q66	175.7	55.7	29.8	121.4	8.37
H67	174.1	55.2	28.9	120.0	8.58
V68	175.7	62.4	32.9	122.5	8.23
I69	175.5	60.9	38.7	125.1	8.28
D70	174.4	52.6	38.3	123.5	8.42
Y71	175.4	58.2	38.8	121.7	8.07
I72	175.9	61.1	38.5	122.6	8.09
L73	176.8	55.3	42.5	125.9	8.13
D74	175.0	52.8	37.9	120.0	8.43
L75		55.4	42.4	123.0	8.13
Q76	176.0	56.1	29.2	120.8	8.27
I77	175.9	61.3	38.8	121.4	7.95
A78	177.6	52.5	19.0	127.5	8.23
L79	177.3	55.3	42.4	121.6	8.14
D80	174.9	52.8	38.1	119.1	8.38
S81	173.9	58.4	63.9	115.9	8.09
H82	172.0	53.3	28.6	120.0	8.38
P83	176.9	63.2	32.2		
T84	174.4	62.1	70.0	116.0	8.37
I85	176.0	60.9	38.8	124.2	8.23
V86	175.9	62.2	32.8	125.0	8.23
S87	174.3	57.9	63.9	119.9	8.32
L88	177.0	55.1	42.5	124.8	8.25

H89	174.2	55.0	28.9	118.7	8.50
H90	174.1	55.2	29.1	120.4	8.61
Q91	175.6	55.8	29.9	122.5	8.55
R92	174.2	54.3	30.0	124.1	8.55
P93	177.3	63.4	32.2		
G94	174.0	45.2		109.3	8.41
Q95	175.8	55.9	29.7	119.6	8.18
N96	175.2	53.4	38.8	120.1	8.54
Q97	175.6	56.0	29.5	121.0	8.36
A98	177.7	52.6	19.2	125.1	8.29
S99	174.5	58.4	63.9	115.2	8.21
R100	176.1	56.1	31.0	123.0	8.35
T101	172.7	60.1	69.7	117.8	8.20
P102	176.6	63.2	32.2		
L103	177.6	55.4	42.4	122.8	8.33
T104	174.6	61.7	70.1	114.8	8.15
T105	174.3	61.7	69.9	116.2	8.13
L106	176.9	55.2	42.6	124.4	8.20
N107	175.4	53.3	38.8	120.1	8.49
T108	174.3	61.9	69.7	114.0	8.08
D109	174.9	53.0	38.0	121.1	8.43
I110	176.0	61.2	38.9	120.6	7.94
S111	174.6	58.3	63.9	119.6	8.29
I112	176.2	61.4	38.6	123.0	8.13
L113	177.4	55.4	42.3	125.2	8.16
S114	174.7	58.4	63.8	116.6	8.19
L115	177.5	55.5	42.3	124.3	8.18
Q116	175.9	56.1	29.4	120.5	8.26
A117	177.7	52.8	19.2	124.8	8.18
S118	174.4	58.3	63.9	114.4	8.13
E119	175.2	55.9	29.0	121.7	8.16
F120	173.8	55.5	39.1	120.6	8.11
P121	176.9	63.4	32.0		
S122	174.8	58.7	63.8	116.0	8.34
E123	175.8	55.9	28.8	122.0	8.34
L124	177.2	55.2	42.3	122.9	8.16
M125	176.3	55.4	33.0	121.3	8.34
S126	174.4	58.4	63.9	116.7	8.28
N127	175.1	53.4	38.8	120.7	8.46
D128	175.2	53.0	38.1	119.3	8.37
S129	174.6	58.8	63.9	116.3	8.19
K130	176.2	56.4	33.1	123.0	8.22
A131	177.5	52.5	19.1	124.7	8.14
L132	177.3	55.3	42.4	121.4	8.14
C133-ox	175.0	55.2	41.6	119.2	8.39
G134	176.1	44.2		111.4	8.36

References

1. Schwarzinger, S.; Kroon, G.J.; Foss, T.R.; Chung, J.; Wright, P.E.; Dyson, H.J. Sequence-dependent correction of random coil NMR chemical shifts. *J. Am. Chem. Soc.* **2001**, *123*, 2970-2978, https://www.ncbi.nlm.nih.gov/pubmed/11457007
2. Schwarzinger, S.; Kroon, G.J.; Foss, T.R.; Wright, P.E.; Dyson, H.J. Random coil chemical shifts in acidic 8 M urea: implementation of random coil shift data in NMRView. *J. Biomol. NMR* **2000**, *18*, 43-48, http://www.ncbi.nlm.nih.gov/pubmed/11061227
3. Wishart, D.S.; Bigam, C.G.; Holm, A.; Hodges, R.S.; Sykes, B.D. ^{1}H, ^{13}C and ^{15}N random coil NMR chemical shifts of the common amino acids. I. Investigations of nearest-neighbor effects. *J. Biomol. NMR* **1995**, *5*, 67-81, https://www.ncbi.nlm.nih.gov/pubmed/7881273
4. Tamiola, K.; Acar, B.; Mulder, F.A. Sequence-specific random coil chemical shifts of intrinsically disordered proteins. *J. Am. Chem. Soc.* **2010**, *132*, 18000-18003, 10.1021/ja105656t.
5. Metzler, W.J.; Constantine, K.L.; Friedrichs, M.S.; Bell, A.J.; Ernst, E.G.; Lavoie, T.B.; Mueller, L. Characterization of the three-dimensional solution structure of human profilin: ^{1}H, ^{13}C, and ^{15}N NMR assignments and global folding pattern. *Biochemistry* **1993**, *32*, 13818-13829, https://www.ncbi.nlm.nih.gov/pubmed/8268157
6. Kiewitz, S.D.; Cabrele, C. Synthesis and conformational properties of protein fragments based on the Id family of DNA-binding and cell-differentiation inhibitors. *Biopolymers (Pept. Sci.)* **2005**, *80*, 762-774,10.1002/bip.20287.
